# Dual oxic-anoxic co-culture enables direct study of anaerobe–host interactions at the airway epithelial interface

**DOI:** 10.1128/mbio.01338-24

**Published:** 2025-04-09

**Authors:** Patrick J. Moore, Kayla Hoffman, Sara Ahmed, Joshua R. Fletcher, Talia D. Wiggen, Sarah K. Lucas, Sabrina J. Arif, Adam J. Gilbertsen, Leslie A. Kent, Jessica K. Fiege, Ryan A. Langlois, Scott M. O'Grady, Ryan C. Hunter

**Affiliations:** 1 Department of Microbiology and Immunology, University of Minnesota 205104 , Minneapolis, Minnesota, USA; 2 Department of Microbiology and Immunology, SUNY at Buffalo 192705, Buffalo, New York, USA; 3 Department of Population Health and Pathobiology, North Carolina State University 6798 , Raleigh, North Carolina, USA; 4 Department of Biology, Syracuse University 171413, Syracuse, New York, USA; 5 Department of Animal Science, University of Minnesota 311836 , Saint Paul, Minnesota, USA; University of Colorado Anschutz Medical Campus, Aurora, Colorado, USA

**Keywords:** *Fusobacterium nucleatum*, airway epithelium, RNAseq, single-cell RNA sequencing

## Abstract

Strict and facultative anaerobic bacteria are widely associated with both acute and chronic airway diseases. However, their potential role(s) in disease pathophysiology remains poorly understood due to inherent limitations of existing laboratory models and conflicting oxygen demands between anaerobes and host cells. To address these limitations, here, we describe a dual oxic-anoxic culture (DOAC) approach that maintains an oxygen-limited microenvironment at the apical epithelial interface while host cells are oxygenated basolaterally. This platform enables epithelial–anaerobe co-culture for ~48 h, and we demonstrate its utility by evaluating reciprocal interactions between the oxygen-sensitive anaerobic bacterium, *Fusobacterium nucleatum,* and oxygen-demanding airway epithelial cells at the transcriptional level. Using bulk RNAseq, we demonstrate that epithelial colonization results in altered gene expression by *F. nucleatum,* highlighted by the differential expression of genes associated with virulence, ethanolamine and lysine metabolism, metal uptake, and other transport processes. We also combine DOAC with single-cell RNA sequencing to reveal a cell type-specific transcriptional response of the airway epithelium to *F. nucleatum* infection, including the increased expression of inflammatory marker genes and cancer-associated pathways. Together, these data illustrate the versatility of DOAC while revealing new insights into anaerobe–host interactions and their mechanistic contributions to airway disease pathophysiology.

**IMPORTANCE:**

Conflicting oxygen demands between anaerobes and host cells present a significant barrier to *in vitro* modeling of how these cell types interact. To this end, the significance of our dual oxic-anoxic culture (DOAC) approach lies in its ability to maintain anaerobe and epithelial viability during co-culture, paving the way for new insights into the role(s) of anaerobic microbiota in disease. We use DOAC to interrogate reciprocal interactions between the airway epithelium and *Fusobacterium nucleatum*—an anaerobic commensal with pathogenic potential. Given its link to a range of diseases, from localized infections to various cancers, these data showing how *F. nucleatum* bacterium re-shapes its metabolism and virulence upon epithelial colonization provide new mechanistic insight into *F. nucleatum* physiology and how the host responds. We use *F. nucleatum* as our model, but the DOAC platform motivates additional studies of the gut, lung, and oral cavity, where host–anaerobe interactions and the underlying mechanisms of pathogenesis are poorly understood.

## INTRODUCTION

Decades of clinical laboratory culture have focused on a limited set of pathogens associated with acute and chronic airway disease (e.g., *Pseudomonas aeruginosa, Staphylococcus aureus*). More recently, culture-independent sequencing studies of airway microbiota have identified complex bacterial signatures, lending evidence to polymicrobial disease etiologies. Notably, oral-associated facultative and obligate anaerobes*—Fusobacterium, Prevotella, Veillonella, Streptococcus* spp.—which are present at low densities in the healthy respiratory tract (1, 2), are both prevalent and abundant in chronic obstructive pulmonary disease (COPD), cystic fibrosis (CF), non-CF bronchiectasis, sinusitis, ventilator-associated pneumonia, and other respiratory complications (3–8). In each case, the development of hypoxic microenvironments at the airway epithelial interface creates a niche for anaerobe proliferation, often reaching densities equal to or greater than those of canonical pathogens.

Mechanistic contributions of anaerobic bacteria to airway disease remain poorly understood, though several roles have been proposed. In healthy individuals, anaerobe abundance in bronchoalveolar lavage fluid correlates with expression of pro-inflammatory cytokines, elevated Th17 lymphocytes, and a blunted toll-like receptor 4 (TLR4) response, implicating a compromised first line of defense against bacterial infection (1). Indeed, epidemiologic and *in vitro* data suggest that anaerobes may facilitate secondary colonization by canonical airway pathogens. In non-CF bronchiectasis, *Prevotella* and *Veillonella* positively correlate with Th17 cytokines and non-tuberculosis mycobacterial infection (9). Similarly, in HIV subjects, anaerobes suppress expression of interferon gamma and interleukin-17A (IL-17A) via production of short-chain fatty acids (SCFAs) and are thought to impair the host response to consequent *Mycobacterium tuberculosis* colonization (7). In CF, anaerobe-derived SCFAs increase with age and disease progression (10), mediate excessive production of IL-8 by bronchial epithelial cells (in turn promoting neutrophil mobilization) (11), and can potentiate the growth and virulence of canonical airway pathogens (12, 13). Anaerobe-dominated bacterial communities in the CF airways are more commonly associated with milder disease (14), but they also increase in abundance during acute disease flares prior to antibiotic therapy (15), implicating anaerobes in pathogenesis.

Direct studies of anaerobe–host and anaerobe–host–pathogen interactions have been limited by the lack of suitable laboratory models. Animal models often poorly reflect chronic infection pathologies and/or can be prohibitively expensive for high-throughput analyses. As an alternative, three-dimensional (3D) cell culture approaches have greatly expanded our knowledge of microbial–epithelial interactions (16, 17). However, incorporation of anaerobic microbiota into these models is restricted by the inherent challenge of maintaining host cell viability under oxygen-limited culture conditions. Newer microfluidic-based and organ-on-a-chip approaches have also seen widespread interest for establishing hypoxic or anoxic epithelial interfaces, but these can be technically demanding and require specialized instrumentation (18–20). Newer, more accessible, and versatile *in vitro* approaches are needed for a more detailed understanding of anaerobic microbiota and their potential roles in chronic airway disease.

Here, we describe a dual oxic-anoxic culture (DOAC) approach that recapitulates an oxygen-limited epithelial microenvironment thought to exist in the diseased airways (21). In this model, polarized epithelial monolayers are maintained at air–liquid interface (ALI) in an anaerobic chamber, while O_2_ and CO_2_ are delivered to the culture apparatus from an external source. This setup allows for maintenance of hypoxia on the apical surface (akin to chronic airway infection) while host cells are oxygenated basolaterally. To demonstrate the utility of the model, we combine DOAC with fluorescence *in situ* hybridization and bulk RNA sequencing to evaluate epithelial colonization and the transcriptional response of *Fusobacterium nucleatum*—an anaerobic oral commensal with pathogenic potential (22). We also combine DOAC with single-cell RNA sequencing (scRNAseq) to reveal the cell type-specific transcriptional response of normal human tracheal bronchial epithelial (nHTBE) cells to *F. nucleatum* infection, including the increased expression of inflammatory marker genes and pathways involved with cell adhesion, invasion, proliferation, and repair. By revealing reciprocal transcriptional interactions between anaerobe and host, we not only demonstrate the power and versatility of the DOAC platform but also offer new insights into potential roles of an anaerobic opportunist in the onset and development of airway disease.

## RESULTS

### Optimization of a dual oxic-anoxic epithelial culture platform

**Fig 1:**
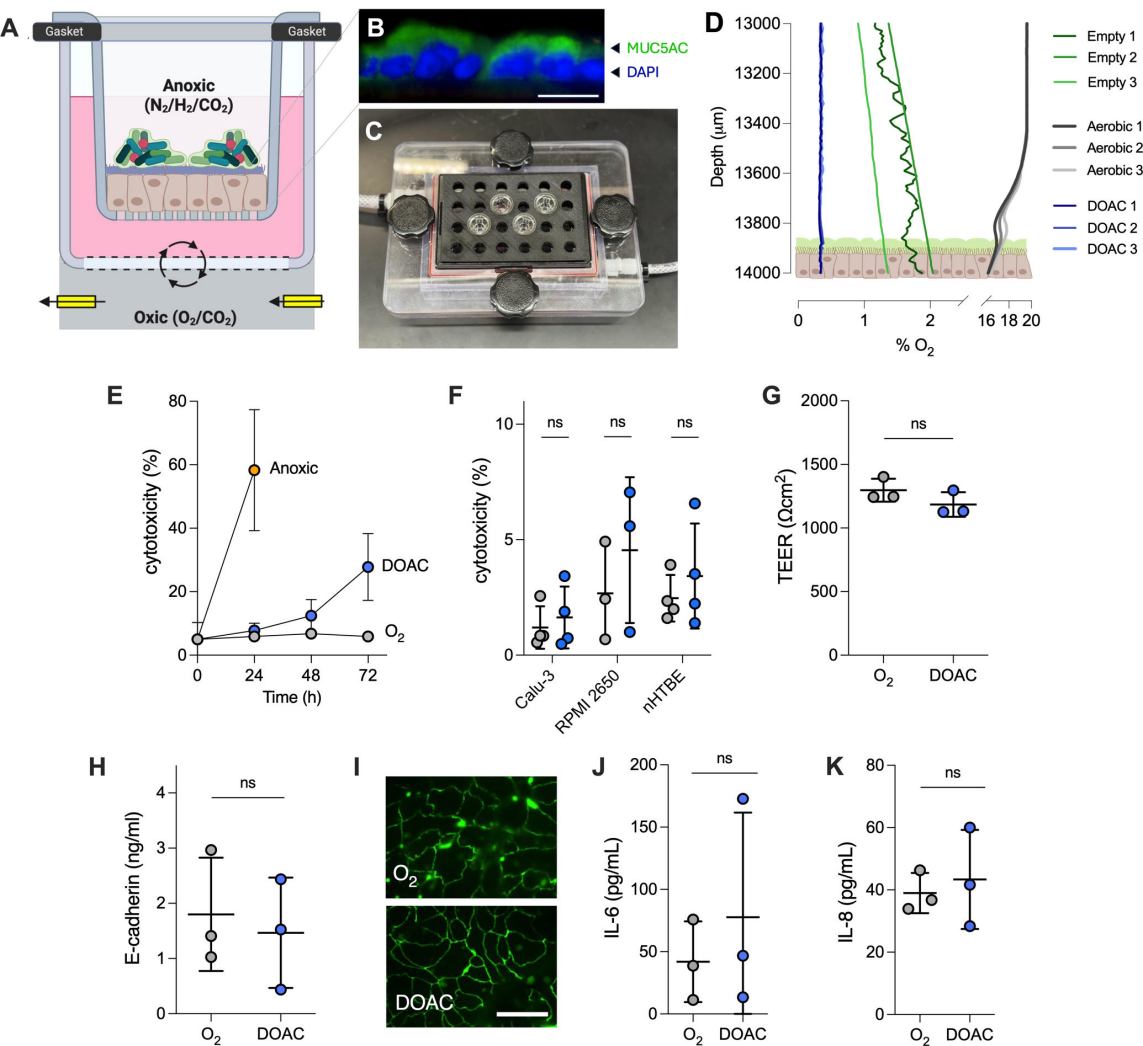
Optimization of a dual oxic-anoxic epithelial culture platform. (**A**) Primary and immortalized (Calu-3, RPMI 2650) epithelial cells are cultured on Transwell inserts at ALI for 21–28 days, transferred to a gas-permeable multi-well plate manifold in an anaerobic chamber, and incubated under dual oxic-anoxic culture conditions where the apical compartment is exposed to the oxygen-limited environment of the chamber and mixed blood gas (21% O_2_, 5% CO_2_, 74% N_2_) is delivered to the basolateral compartment to oxygenate the epithelial cells. Figure created with BioRender.com. (**B**) Calu-3 cells produce a confluent mucus layer on the apical surface, mimicking chronic airway disease (blue, 4',6-diamidino-2-phenylindole (DAPI); green, anti-MUC5AC; bar = 25 µm). (**C**) Transwells are mounted in a custom 3D-printed thermoplastic polyurethane gasket mounted on a gas-permeable multi-well plate (see also Fig. S1). Blood gas is delivered and removed through cable glands mounted to the basolateral compartment of the Transwell-containing apparatus. (**D**) Representative oxygen microprofiles. Under normoxic growth conditions (grayscale), Calu-3 cells exhibit a slight oxycline at the epithelial surface. DOAC cells (blue) maintain an anoxic epithelial microenvironment below the level of detection (<0.3%). Empty Transwells in the DOAC system (green) show a gradual oxycline above the Transwell membrane. Profiles represent *n* = 3 biological replicates. (**E**) Cytotoxicity as measured by lactate dehydrogenase (LDH) in the DOAC medium over time relative to normoxic (O_2_^+^) and strict anoxic (O_2_^−^) culture conditions. LDH activity is expressed as a percent relative to normoxic cultures at *t* = 0 and a lysed control. (**F**) LDH release by Calu-3, RPMI 2650, and primary nHTBE cells under normoxic culture (gray) and DOAC (blue) conditions after 24 h. (**G**) Transepithelial electrical resistance (TEER), (**H**) E-cadherin concentrations, (**I**) immunofluorescence of zonula occludens-1 (ZO-1) tight junction proteins (bar = 20 µm), and cytokines (**J**) IL-6 and (**K**) IL-8 showed no significant differences between normoxic culture and DOAC conditions after 24 h. All data shown for panels E–H, J, and K were derived from at least three independent experiments with three technical replicates each and were compared using a Mann-Whitney U-test. Bars in each panel represent the mean ± SD.

Our primary objective was to develop and optimize a culture platform that facilitates co-culture of anaerobic bacteria and airway epithelial cells (Fig. 1A through C; Fig. S1). To do so, we first grew polarized monolayers of the adenocarcinoma cell line, Calu-3, at ALI in Transwell inserts for 21–28 days under standard (normoxic) conditions. Polarized Calu-3s produce a distinct mucus layer on the apical surface (Fig. 1B), mimicking aberrant mucin accumulation associated with CF, COPD, sinusitis, and other chronic airway diseases. Once polarized, Transwells are placed in a custom 3D-printed thermoplastic polyurethane gasket mounted on a 24-well gas-permeable culture plate and transferred to a culture chamber housed in an anaerobic workstation (Fig. 1C; Fig. S1). While in the chamber, mixed blood gas (21% O_2_, 5% CO_2_, 74% N_2_) is delivered and removed through cable glands mounted to the basolateral compartment of the Transwell-containing apparatus (Fig. 1A; Fig. S1). The goal of this DOAC setup was to achieve oxygenation of host cells while maintaining exposure of the apical epithelial surface to the oxygen-limited environment of the anaerobic chamber, facilitating the growth of anaerobic bacteria.

To first determine whether a strict anaerobic environment was achieved at the apical surface, we used fiber-optic oxygen microsensors to profile O_2_ concentrations throughout the Transwell under normoxic and DOAC conditions (Fig. 1D; Fig. S2). In contrast to ambient air (normoxia) where O_2_ concentrations drop from 21% (saturation) to ~16% oxygen in the Calu-3 mucus layer, oxygen remained below the limit of detection (0.3%) at the epithelial surface when cultured under DOAC conditions. In empty wells that did not contain epithelial cells, a gradual oxycline was detectable several hundred microns above the Transwell membrane while housed in the anaerobic chamber, confirming that a confluent epithelial layer is sufficient to restrict oxygen diffusion into the apical environment while cultured in the DOAC system.

To further evaluate the performance of DOAC, we determined that, when compared to cell culture under normoxic and strict anoxic conditions (i.e., no O_2_ delivery), Calu-3 cytotoxicity was negligible up to 24 h as determined by lactate dehydrogenase (LDH) release, with a progressive increase in cell death after 48 h and 72 h (Fig. 1E). Viability of the immortalized RPMI 2650 nasal epithelial cell line and primary human tracheal bronchial epithelial cells was similarly maintained under DOAC conditions for at least 24 h (Fig. 1F)**,** supporting its use for a range of epithelial cell types. We then determined the effects of DOAC, if any, on Calu-3 cell physiology after 24 h, which we selected as the time point for downstream experiments. Transepithelial electrical resistance (TEER) (Fig. 1G) and E-cadherin concentrations (Fig. 1H), both proxies of epithelial barrier integrity, showed no differences between DOAC conditions and normoxic controls. These data were corroborated by immunofluorescence microscopy which revealed confluent monolayers and well-defined staining of tight junction zonula occludens (ZO) proteins that appeared as near-continuous rings at the periphery of each cell (Fig. 1I). Previous work has shown that hypoxia induces expression of pro-inflammatory cytokines in pulmonary fibroblasts (23). Thus, we also used an enzyme-linked immunosorbent assay to quantify IL-6 and IL-8 production. Both cytokines showed no significant increases under DOAC conditions relative to normoxia (Fig. 1J and K), demonstrating that reduced apical oxygen does not elicit an inflammatory response in Calu-3 cells after 24 h.

**Fig 2:**
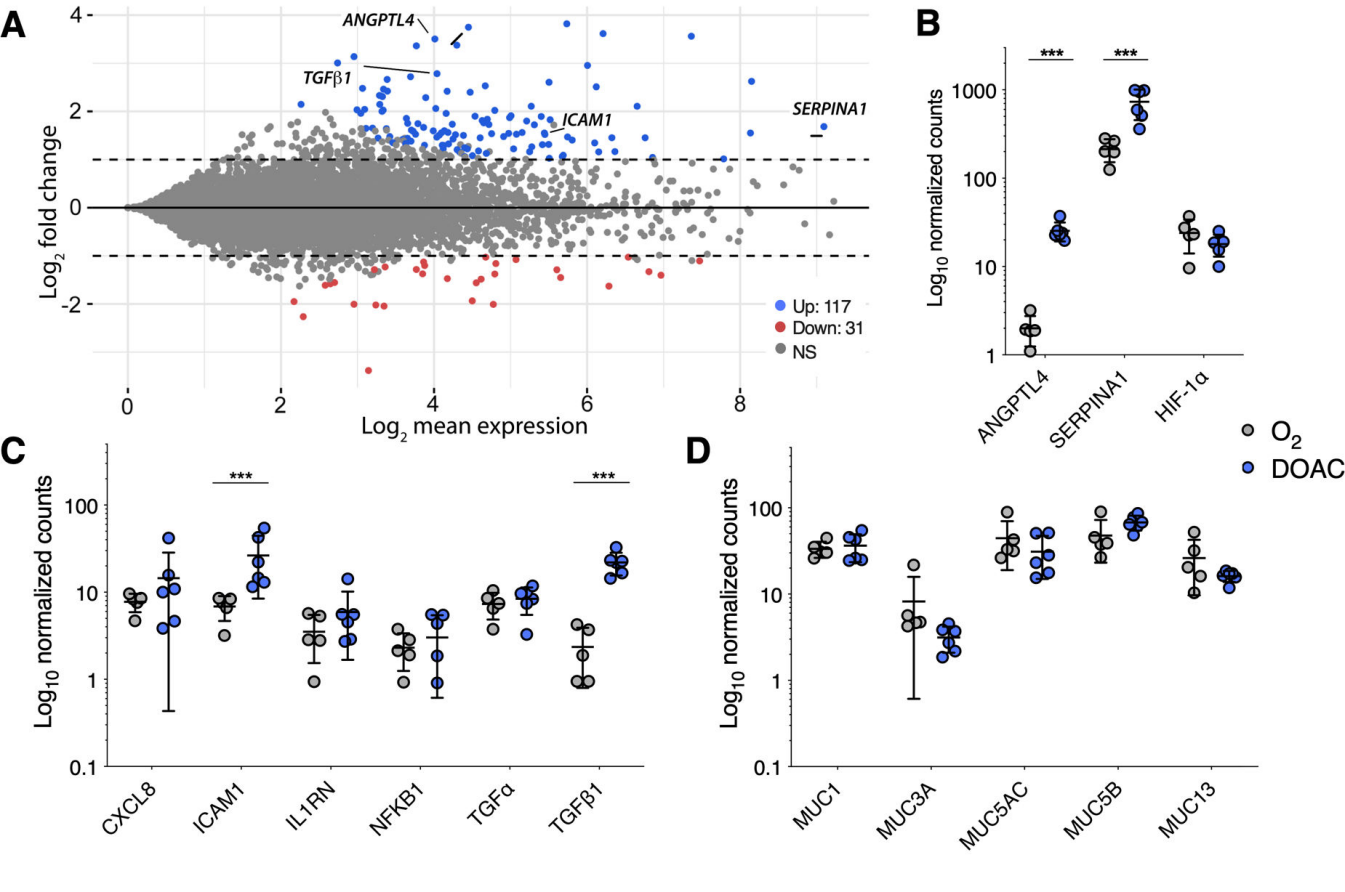
Dual oxic-anoxic culture of Calu-3 cells yields a similar transcriptomic profile to normoxic culture conditions (ALI). (**A**) Log ratio/mean average (MA plot) representation of Calu-3 gene expression under DOAC conditions relative to normoxic culture at ALI (117 upregulated [blue], 31 downregulated [red], log2fc > 1, *P*adj <0.001). (**B**) *ANGPTL4* and *SERPINA1* were differentially expressed, though HIF-1α was consistent between cultures. Few changes in (**C**) inflammatory biomarkers and (**D**) mucin gene expression were observed. Data shown in panels B–D are log_10_-normalized gene counts from five or six independent biological replicates and were compared using the Wald test, Benjamini-Hochberg adjusted (***, *P* < 0.001).

To gain a more comprehensive understanding of the physiological response of Calu-3 cells to dual oxic-anoxic culture, we used bulk RNAseq to compare global Calu-3 gene expression to culture under normoxic conditions. After 24 h, transcriptome analysis revealed 148 differentially expressed transcripts (117 upregulated, 31 downregulated, log_2_ fold change [l2fc] ≥ 1, *P*adj < 0.001) out of ~16,000 total genes (Fig. 2A; Data set S1) (1). In contrast, after 48 h, 3,737 genes were differentially expressed (2,816 up, 921 down) coinciding with increased cytotoxicity at that later time point (Fig. S3; Data set S2). Aside from *ANGPTL4* (encoding angiopoietin-like 4) and *SERPINA1* (alpha-1 antitrypsin) (Fig. 2B), which are induced in response to hypoxia and acute inflammation, respectively, few markers of cell stress were differentially expressed at 24 h, including genes involved in tight junction formation, oxidative stress, and endoplasmic reticulum stress. Importantly, HIF-1α, which is constitutively expressed at low levels under normoxia but upregulated under hypoxia, was also consistent between atmospheres after 24 h, suggesting that Calu-3 cells are sufficiently oxygenated under DOAC conditions (Fig. 2B). Among inflammatory biomarkers, only *ICAM1* (intracellular adhesion molecule 1) and *TGF*β*1* (transforming growth factor beta 1) showed statistically significant differences, further demonstrating that DOAC did not yield an appreciably inflammatory microenvironment (Fig. 2C). Finally, since we use the DOAC model to model bacterial colonization of the apical mucus layer, we compared mucin-related gene expression between conditions. Among detectable transcripts (*MUC1, MUC3A, MUC5AC, MUC5B,* and *MUC13*), no significant differences were observed between culture conditions (Fig. 2D). These data demonstrate that DOAC yields some changes in cytotoxicity and gene expression, but we consider these changes to be negligible relative to the many advantages of the DOAC platform for modeling interactions between anaerobic microbiota and the host.

### DOAC enables anaerobe–epithelial co-culture

As described above, the lack of tractable epithelial cell culture systems compatible with hypoxic or anoxic bacterial growth has limited our understanding of anaerobe–host interactions. Prior work has shown that culture supernatants of anaerobic bacteria elicit pro-inflammatory cytokine expression by airway epithelial cells *in vitro* through the production of SCFAs (1, 10, 11). However, it is not yet known how the host responds to the direct presence of anaerobes at the epithelial interface, nor how anaerobic microbiota alter their behavior in response to the epithelial microenvironment. To address this knowledge gap, we used DOAC to evaluate interactions between Calu-3 cells and *Fusobacterium nucleatum*—an obligately anaerobic pathobiont. This bacterium is commonly found in both healthy and disease states of the oral cavity, though it can also colonize extra-oral sites including the airways, where it has been linked to pathogenesis in COPD, CF, sinusitis, aspiration pneumonia, empyema, and other complications (13, 24–26). Given its link to inflammatory bowel disease, colorectal cancer, and gastroesophageal reflux disease, it was chosen as a broadly relevant bacterium with which to test the DOAC system.

**Fig 3:**
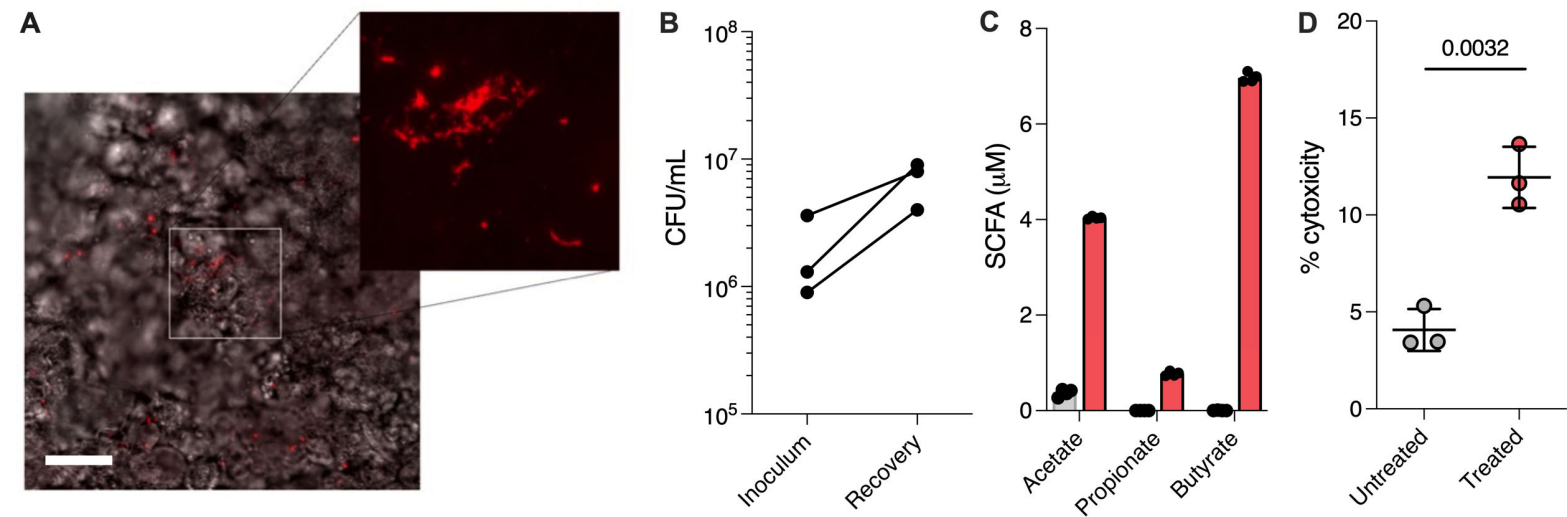
DOAC enables *F. nucleatum* colonization of and co-culture with Calu-3 epithelial cells. (**A**) After 24 h of DOAC co-culture, HCR-FISH imaging was used to confirm and visualize *F. nucleatum* colonization of Calu-3 cells and aggregation at the apical interface. Bar = 50 µm. (**B**) After 24 h of co-culture, *F. nucleatum*-treated Calu-3 cells were resuspended with PBS, and bacterial recovery relative to the inoculum was quantified by plating on selective agar. (**C**) SCFA quantification in spent culture media from challenged Calu-3 cells (blue) relative to untreated controls (gray). (**D**) *F. nucleatum* exhibited a slight cytotoxic effect after 24 h. Data shown in panel C represent the mean ± SD of four independent experiments. Data in panel D represent the mean ± SD of three independent experiments with three technical replicates for each condition and were compared using a Mann-Whitney U-test.

After 3 h of equilibration under DOAC conditions, polarized Calu-3 cells were challenged with ~2 × 10^6^ colony-forming units (CFU) of *F. nucleatum* subsp. *nucleatum* ATCC 25586 and incubated in the anaerobic chamber for an additional 24 h. Since fluorescent markers are limited for *F. nucleatum,* we used hybridization chain reaction (HCR) fluorescence *in situ* hybridization (FISH) imaging to visualize its colonization of the apical interface. HCR relies on hybridizing a target 16S rRNA (or mRNA transcript) with nucleic acid probes that trigger amplification of fluorescently labeled DNA hairpins into polymer chains, enabling multiplexed mapping of multiple target RNAs at small spatial scales (27). After 24 h, co-cultures were chemically fixed, and Transwell membranes were removed from their plastic casing. Membranes were HCR labeled and visualized using epifluorescence microscopy to confirm bacterial colonization (Fig. 3A; Fig. S4). Here, *F. nucleatum* formed a heterogeneous surface-attached arrangement that ranged between individual cells and densely packed cell clusters, likely reflecting their aggregative phenotype (28). Interestingly, we did not observe epithelial invasion, which is also a well-known *F. nucleatum* behavior in the gastrointestinal tract (29). Importantly, viable bacterial cells (7 × 10^6^ ± 2 × 10^6^) were recovered after 24 h by washing the apical surface with phosphate-buffered saline (PBS) and plating on selective medium (Fig. 3B). The microbial-derived short-chain fatty acids acetate (4.0 µM), butyrate (7.0 µM), and propionate (0.8 µM) were also detectable in the culture medium after 24 h but not in uninfected control cultures, confirming that apical oxygen concentrations were sufficiently low for anaerobe viability and fermentative metabolism (Fig. 3C). We note that in contrast to the canonical airway pathogen *S. aureus,* which is completely cytotoxic to Calu-3 cells during co-culture (30), only a slight (but statistically significant) cytotoxic effect was induced by *F. nucleatum* colonization (Fig. 3D).

### *F. nucleatum* alters its global transcriptional profile during anaerobic co-culture with Calu-3 cells

**Fig 4:**
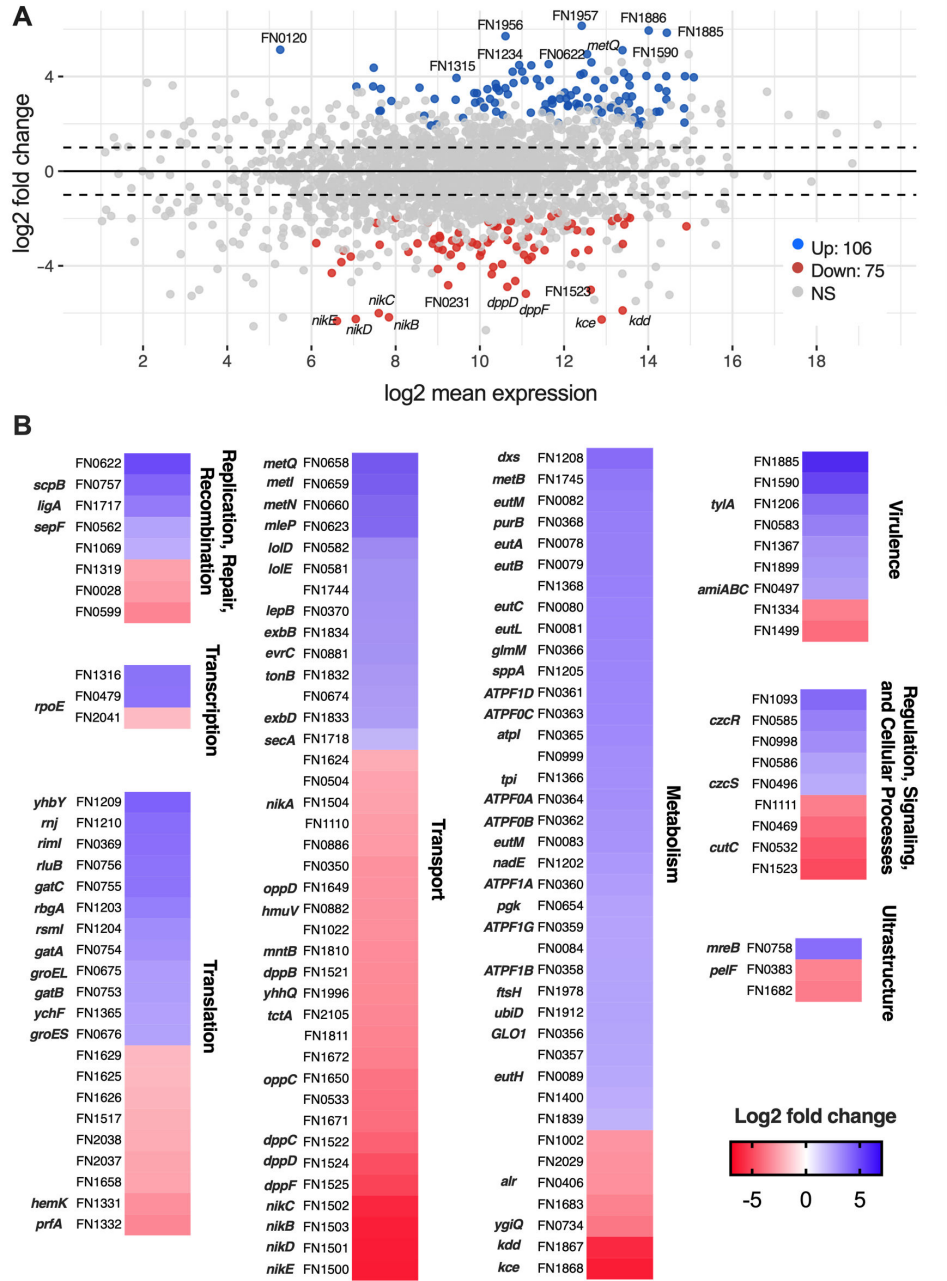
*F. nucleatum* alters its global transcriptional profile during anaerobic co-culture with Calu-3 cells. (**A**) Log ratio/mean average (MA plot) representation of *F. nucleatum* differential gene expression during DOAC co-culture with Calu-3 epithelial cells relative to planktonic culture in EMEM. (l2fc > 1; *P*adj < 0.001). (**B**) Heat maps depicting fold change gene expression in specific *F. nucleatum* genes by functional category (KEGG orthology). Genes encoding hypothetical proteins and those of unknown function are not shown (see Table S1).

Given that *F. nucleatum* differentially regulates its expression of genes involved in amino acid metabolism, protein export, and virulence during Caco-2 epithelial invasion (31), we reasoned that *F. nucleatum* would express a unique transcriptional profile in response to colonization of the Calu-3 interface. Thus, to gain insight into genes and pathways that are differentially regulated upon airway epithelial colonization relative to planktonic growth, Calu-3-containing Transwells cultured under DOAC conditions were challenged with *F. nucleatum* for 24 h. Planktonic cultures in Eagle’s minimal essential medium (EMEM) were grown in parallel. Total RNA was then extracted from Calu-3 co-cultures and planktonic controls and was subjected to ribodepletion prior to Illumina sequencing. Sequence reads were mapped to the *F. nucleatum* subsp. *nucleatum* ATCC 25586 genome using RSubread (32). Differential gene expression between culture conditions was then performed using DESeq2 (33) (Fig. 4; Table S1; Data set S3).

As predicted, 181 genes were differentially expressed during epithelial colonization relative to planktonic culture (117 up, 31 down, l2fc ≥ 1, *P*adj < 0.001, >10 mapped sequence reads per gene). An MA plot representing this analysis is shown in Fig. 4A, annotated with 20 of the most highly differentially expressed genes (10 upregulated, 10 downregulated). Among genes exhibiting the greatest fold change in expression was FN1885, predicted to encode hemolysin III—a virulence protein with 96% identity to a hemolysin (FSDG_RS08715) in *Fusobacterium animalis* previously shown to be upregulated upon invasion of the intestinal epithelium (31). Other top upregulated genes included FN1590 (putative virulence lipoprotein) (34), FN0622 (DNA lyase), FN0658 (ABC transporter substrate-binding protein MetQ), and six genes encoding hypothetical proteins. For these latter six, further exploration using paperBLAST (35) and HMMER (36) uncovered no known homologs.

Top downregulated loci included four genes (FN1500, FN1501, FN1502, FN1503) of the *nik* operon, encoding a periplasmic-binding-protein-dependent transport system. Disruption of analogous genes in *Escherichia coli* and *Proteus mirabilis* greatly reduces nickel-containing enzyme activity and virulence (37, 38). FN1868 (*kce) and* FN1867 (*kdd*)*,* encoding a 3-keto-5-aminohexanoate cleavage enzyme and l-erythro-3,5-diaminohexanoate dehydrogenase, respectively, were also downregulated, likely reflecting a switch from lysine fermentation during planktonic growth to alternate metabolic processes and pathways for butyrate production upon colonization (39). FN1525 (*dppF*) and FN1524 (*dppD*)*,* which both encode ATP binding components of a dipeptide ABC transporter complex, were likewise among the top 10 downregulated genes, as were a putative diguanylate phosphodiesterase (FN1523) and a hypothetical protein (FN0231) with no known function.

An expanded look at the DESeq2 data (Fig. 4B) revealed additional insights into *F. nucleatum* behavior upon colonization. Based on KEGG orthology and grouping based on basic functional category, many differentially expressed genes were linked to central metabolism and transport. Among genes exhibiting increased expression were several associated with ethanolamine (EA) catabolism, including *eutB* (FN0079) and *eutC* (FN0080), both of which encode subunits of EA ammonia lyase that breaks EA down into ammonia and acetaldehyde (40). The latter is further converted to acetyl CoA via acetaldehyde dehydrogenase (FN0089, which also increased in expression), and is consistent with the production of short-chain fatty acids in the spent medium. Other notable upregulated processes included methionine transport and utilization (FN0658–FN0660, FN1745), several regulatory proteins including a *czcRS*-like two-component system (FN0585, FN0586) linked to metal homeostasis (41), *tylA* (FN1206, encoding a putative hemolysin), and other virulence-associated lipoproteins (FN0583, FN1899), and the co-chaperonins *groEL* (FN0675) and *groES* (FN0676). Interestingly, in addition to the *nik* and *dpp* operons, a third ABC transport system (*OppABCDF*) involved in the uptake of oligopeptides (42) was also notably repressed. Altogether, we interpret this pattern of regulation to suggest that *F. nucleatum* responds to epithelial colonization by shifting its metabolism and transport processes to optimize use of bioavailable nutrient sources (e.g., ethanolamine, trace metals) while increasing expression of signaling and virulence pathways that may contribute to its pathogenicity.

### Single-cell RNA sequencing reveals cell-specific expression of inflammatory marker genes and cancer-related pathways in response to *F. nucleatum*

A drawback to transcriptomic studies of epithelial cell populations is that bulk RNA sequencing necessarily averages out contributions of different host cell types to the overall transcriptional landscape. Not only does this mask how physiological processes are distributed among cells, but contributions from rare cell types are also largely invisible. To address these limitations, scRNAseq has been widely adopted in respiratory research and has already led to the discovery of ionocytes and novel insights into the immune landscape of lung cancer, mononuclear phagocytes in the CF lung, and the host response to severe acute respiratory syndrome coronavirus 2 infection (43–46). To our knowledge, scRNAseq has not yet been applied to bacterial–host interactions in the context of airway disease. Given the many examples of tissue and cell-specific tropism exhibited by pathogens of the respiratory tract (47–49), here, we combined DOAC with scRNAseq to test our hypothesis that *F. nucleatum* elicits an inflammatory host response that varies by epithelial cell type.

**Fig 5:**
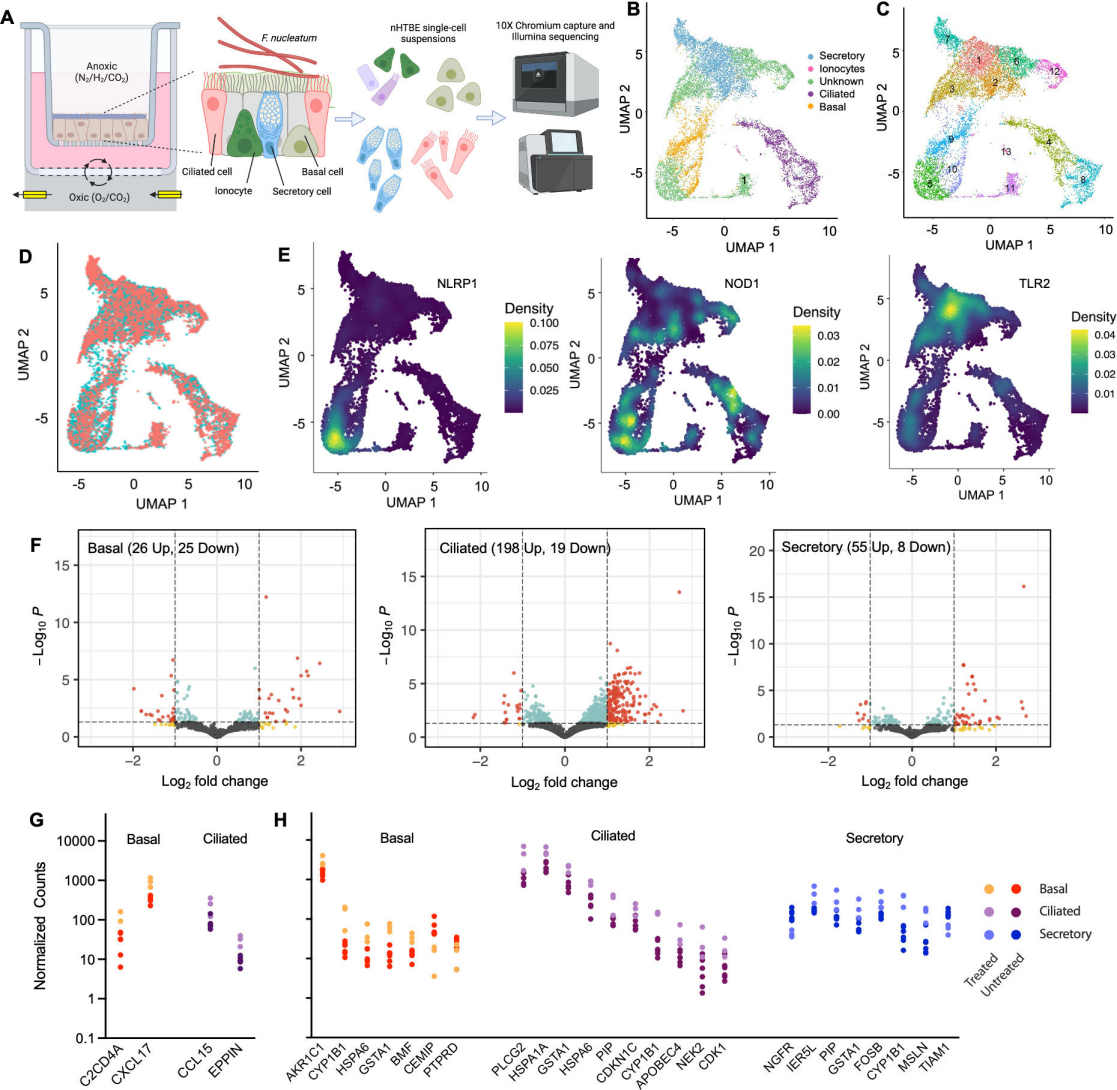
Single-cell RNA sequencing of *F. nucleatum*-treated nHTBE cells reveals a cell type-specific transcriptional response. (**A**) Experimental schematic. Primary airway epithelial cells were cultured under DOAC conditions and challenged with *F. nucleatum*. Host cells were dissociated and collected via the 10X Genomics platform prior to scRNAseq. (**B**) UMAP projection of nHTBEs showing four major cell types (and unknown cells) from (**C**) 13 distinct clusters. (**D**) UMAP projection of nHTBE cell types by treatment condition. (**E**) Density plots (Nebulosa projections) of NLRP1, NOD1, and TLR2 showing heterogeneous expression of pattern recognition receptors between cell types. (**F**) Volcano plot representation of differential gene expression in basal, ciliated, and secretory cells between *F. nucleatum*-treated nHTBE cultures relative to untreated controls. (red, log_10_ expression >1 and l2fc > 1; blue, log_10_ expression >1 only; yellow, l2fc > 1 only). (**G, H**) Scatterplot representations of differentially expressed (**G**) inflammatory markers and (**H**) pathways associated with cell migration, proliferation, and migration, arranged by cell type. scRNAseq data are derived from at least three independent replicates for each condition (untreated, *n* = 6; treated *n* = 3).

Primary human bronchial epithelial (nHTBE) cells were cultured with Pneumacult media at ALI under normoxia, which we evaluated using flow cytometry to confirm differentiation into ciliated, basal, and secretory (including goblet) cells (Fig. S5). After ~21 days at ALI, cell cultures were transferred to DOAC conditions for 3 h, followed by apical challenge with *F. nucleatum*. After 24 h of co-culture, we captured nHTBEs using the 10X Genomics Chromium controller and sequenced an average of 2,007 cells per untreated replicate (*n* = 6) and 3,682 from each *F. nucleatum-*treated replicate (*n* = 3) (Fig. 5A). After removing cells with less than 250 detectable genes and additional filtering criteria, we analyzed a total of 6,981 untreated and 5,010 treated cells, with an average of 4,042 and 3,103 expressed genes per cell, respectively. To identify main cell types, we performed a combined analysis of all cells from all samples using uniform manifold approximation and partitioning (UMAP) (50). This analysis partitioned cells into 13 distinct clusters based on gene expression profiles (colored by cell identity in Fig. 5B) which did not appreciably differ between treated and untreated sample sets (Fig. 5D). The identity of each cluster was determined *post hoc* by identifying genes significantly upregulated within each cluster and by comparison to established sets of marker genes for canonical epithelial cell types (Fig. S6) (43). Consistent with our flow cytometry data, this analysis also identified discrete subpopulations of each cell type (e.g., unique secretory clusters) that showed unique transcriptional profiles (Fig. 5C; Fig. S7). Among clusters, we observed cell type-specific expression of 17 pattern recognition receptors (PRRs) that play a critical role in the innate host response to microbial encounter (Fig. 5E; Fig. S8). For example, TLR2 was predominantly expressed by secretory cells, while NOD1 and NLRP1 exhibited higher expression in ciliated and basal cells, respectively. These data are consistent with previous reports describing heterogeneous expression of PRRs across airway epithelial cell types (51) and supported our hypothesis that the host response to bacterial challenge would also be cell type specific.

Using DESeq2, we performed differential gene expression analysis, accounting for both the effects of cell type and bacterial challenge. As predicted, comparison of *F. nucleatum-*treated cells to untreated controls revealed a distinct, cell type-specific response to bacterial challenge: basal, 34 up/21 down; ciliated, 217 up/23 down; and secretory cells, 77 up/10 down (*P*adj < 0.001) (Fig. 5F; Data set S4). Among notable differentially expressed genes, *F. nucleatum* stimulated expression of multiple cytokines and chemokines, including *CCL15* in ciliated cells and CXCL17 in basal cells (Fig. 5G). These data are consistent with previous reports of *F. nucleatum* stimulating inflammatory host responses in the oral cavity and diverse extra-oral body sites (52–54). Unexpectedly, *F. nucleatum* challenge also resulted in a striking differential expression of genes and pathways associated with cell proliferation, invasion, and adhesion (Fig. 5H). For example, *CYP1B1*, a universal cancer marker encoding cytochrome P450 1B1 (55), and *GSTA1* (glutathione S-transferase A1) which promotes lung cell invasion and adhesion (56), increased in expression in each of basal, ciliated, and secretory cells in response to *F. nucleatum* (Fig. 5H). *HSPA1A* (encoding heat shock protein A), involved in the promotion of cell proliferation, metastasis, and invasion (57), was also upregulated in ciliated cells, as was *HSPA6* in basal cells. Twenty-one other cancer-related genes also varied by cell type (*P*adj < 0.001). While it is possible that these transcriptional profiles and their heterogeneous expression between cell types are consistent with a direct role for *F. nucleatum* in carcinogenesis (58, 59), the altered expression of these genes may also reflect tissue remodeling and repair in response to bacterial challenge. To our knowledge, these data are the first to reveal the airway epithelial response to bacterial challenge with the granularity afforded by scRNAseq and further demonstrate the power of the DOAC platform to model anaerobe–host interactions.

## DISCUSSION

Despite anaerobes comprising a significant component of airway microbiota, studies on their contributions to disease pathophysiology have yielded seemingly contradictory results, calling into question their role(s) in patient morbidity. Proposed pathogenic mechanisms are supported by compelling *in vitro* data (10–12), but their relevance is unresolved due to a lack of compatible models with which to test their interactions with the respiratory epithelium. To address this knowledge gap, here, we describe a model platform (DOAC) that enables co-culture of obligate anaerobes with polarized airway epithelial cells. Importantly, provision of oxygen exclusively to the basolateral side of host cells prevented anoxia-associated cytotoxicity, inflammation, and significant changes in global gene expression, supporting its utility for the study of anaerobe–host interactions. Using this platform, we demonstrate that *F. nucleatum* alters its transcriptional profile upon colonization of the epithelial surface while eliciting a pro-inflammatory and proliferative host response that varies by epithelial cell type.

Disparate oxygen demands between epithelial cells and anaerobic bacteria pose significant challenges for their co-culture *in vitro*. Several culture systems have been developed to overcome these challenges and recapitulate an oxygen-restricted mucosal interface (60–62), though each has limitations. Transwell cultures of Caco-2, HaCaT, and primary human gingival cells have been used to demonstrate that anaerobic taxa can adhere to, invade, and alter oral and intestinal epithelia, yet these assays are either limited to short incubation times or only loosely mimic *in vivo* conditions (63–65). Newer microfluidic-based and “organ-on-a-chip” models have also seen widespread interest due to their ability to establish a dual oxic-anoxic interface and facilitate study of anaerobe–host interactions (18, 19). However, these models either preclude direct host–bacterial contact or are technically challenging to maintain both oxic and anoxic microcompartments. The approach described here (DOAC) offers the advantages of ease of use, direct interactions between host and microbiota, reproducibility given the multi-well plate format, and methodological flexibility lending itself to biochemical, microscopy, and transcriptomic studies. At present, DOAC is limited to ~24–48 h of co-culture potentially due to the accumulation of toxic metabolites, the gradual acidification of the growth medium over time (which we observed), insufficient ATP production, and prolonged anaerobic glycolysis, among other reasons. With the exception of one gene (*PLCG2,* encoding phospholipase C gamma 2) in ciliated cells (Data set S4), we observed no signatures of hypoxia in either stratified primary bronchial epithelial cells or Calu-3 monolayers, suggesting sufficient oxygenation of all cell types. However, we did observe significant upregulation of glucose transport (*SLC2A1*), phosphofructokinase (*PFKFB3*), and enolase 1 (*ENO1*), which are indicative of anaerobic glycolysis, lactate accumulation, and potentially cellular acidosis, ultimately leading to cytotoxicity. We continue to evaluate and address the underlying mechanisms of epithelial cell death through modification of our culture conditions, including but not limited to media replacements, increased gas delivery, and epithelial cell type, with the long-term goal of enabling extended incubations and studies of sustained interactions between the host and anaerobic microbiota.

To demonstrate the utility of the DOAC platform, we first challenged Calu-3 cells, a mucus-overproducing airway epithelial cell line, with *F. nucleatum*. This bacterium is commonly found in the oral cavity where it serves as a bridge organism between early and secondary colonizers, helping to organize and stabilize the oral microbiota. Under disease conditions, it is also highly prevalent at extra-oral sites (66), and emerging evidence suggests its role as a potential pathogen in various conditions. For example, *F. nucleatum* is linked to oral diseases such as gingivitis and periodontitis, where it is thought to exacerbate inflammation, penetrate periodontal tissues, and disrupt the composition of the oral microbiome leading to more severe disease phenotypes (67). More recent studies also implicate its involvement in gastrointestinal complications as it is found in higher abundance and thought to contribute to intestinal inflammation in individuals with inflammatory bowel diseases (68). It has also garnered attention for its association with various cancers of the oral cavity, esophagus, pancreas, breast, and gastrointestinal tract, and evidence suggests that it accelerates tumorigenesis (53), but the exact mechanisms are poorly understood. In the context of chronic airway disease, *F. nucleatum* (among other oral flora) seeds the airways through microaspiration and is recognized as a risk factor in the development of infection in COPD (69), sinusitis (13, 70), and CF (4), where its presence correlates with exacerbations and increased disease severity. Aside from a recent report describing its coaggregation with *P. aeruginosa* to facilitate bacterial invasion and modulation of the epithelial inflammatory response (71), the mechanistic basis of *F. nucleatum*’s role in airway disease is not known. It was this knowledge gap that motivated our choice of this bacterium and airway epithelial cells to highlight the utility of DOAC and investigate their reciprocal interactions in further detail.

As predicted, and consistent with prior studies of *F. nucleatum* gene expression during gastrointestinal (GI) epithelial invasion (31), we observed a considerable change in bacterial gene expression (106 up, 75 down) during the transition from planktonic growth to epithelial colonization, which may reflect its behavior during the initial seeding of the airways. Based on grouping by basic functional categories, colonization appeared to stimulate transport of peptides, amino acids, and inorganic ions, while promoting metabolic diversification, highlighted by the repression of lysine catabolism (*kdd, kce*) and upregulation of genes (FN0078–FN0090) associated with the metabolism of EA. Interestingly, *F. nucleatum* can ferment lysine during the production of butyrate (72), but lysine can also inhibit *F. nucleatum’s* aggregative phenotype with itself and other bacteria of the oral cavity (73). The mechanism of this inhibition is not known, but it is possible that lysine fermentation is repressed to facilitate fusobacterial aggregation and biofilm formation on the host cell surface. EA is a precursor of phosphatidylethanolamine found in most biological membranes, and free EA is known to be present in the GI lumen, in part due to the renewal of the epithelium. Thus, it is likely that upregulation of the *eut* locus is in response to Calu-3 cell turnover or a direct remodeling of the phospholipid layer, which, in the context of disease, could compromise the integrity of the respiratory epithelium. EA can also serve as a signal molecule that modulates expression of virulence factors (40), of which we observed several that are known to increase in expression during epithelial invasion (31), including lipoproteins and hemolysins (FN1955, FN1206, and FN1590) . We did not detect any evidence of intracellular invasion using our HCR imaging approach, but it cannot be ruled out. Finally, we note that genes encoding the canonical virulence factors of *F. nucleatum* (FadA, Fap2, and RadD) exhibited little difference in expression between culture conditions, consistent with previous reports (31, 74).

We elected to test Calu-3 cells as a representative cell line for several reasons. First, Calu-3 cells reach polarization at ALI within ~21 days and achieve TEER values far greater and more stable than those of primary cells (>1,000 Ωcm^2^) (75). In addition, overproduction of mucus on the apical surface of Calu-3 cells mimics a diseased mucosal environment and allowed us to test the hypothesis that mucin degradation enhances pathogen colonization. This was an important consideration as microbial communities, at least in the context of CF and sinusitis, are thought to form within secreted mucus as opposed to the epithelial layer. We also acknowledge the limitations of using Calu-3 cells. Unlike primary cells, which form a pseudostratified epithelium with mucociliary differentiation, Calu-3 cells are derived from human bronchial submucosal glands that comprise a relatively homogenous monolayer. Transcriptional and physiological responses to external stimuli may also be unique to Calu-3s. As an example, *S. aureus* enterotoxin B is known to elicit significant differences in barrier integrity as well as IL-6 and IL-8 production in Calu-3 cells relative to primary tissue (76). These, among other considerations, underscore the importance of using the DOAC platform with additional cell lines and primary tissues to model anaerobe–host interactions in greater detail.

Indeed, the combined use of DOAC, primary bronchial epithelial cells, and scRNAseq revealed a cell-type-specific transcriptional response to *F. nucleatum* challenge. Consistent with the accumulating evidence suggesting its role in the progression of various cancers (77), data presented here likewise suggest a pro-inflammatory and potentially tumorigenic effect of *F. nucleatum* on the airway epithelium. Recent work demonstrated that *Fusobacterium* spp., among other oral microbiota, correlate with lower airway inflammation and are enriched in advanced lung cancers (58, 59). However, to our knowledge, a direct effect of *F. nucleatum* on proliferative, invasive, and repair pathways in the airways was previously unknown, as was the cell-type-specific transcriptional response of the host. Whether *F. nucleatum* has a colonization preference for specific cells over others requires further study, but the unique distribution of PRRs between basal, secretory, and ciliated cells insinuates that detection of the bacterium and/or its secreted metabolites is at least partially responsible for the heterogeneous host response. If immunomodulatory therapies are to be further developed and used clinically, understanding which bacterial species promote or mitigate inflammation and how individual cells respond will be critical information to have in hand. The DOAC platform paves the way for further study of these and other aspects of the host–anaerobe dynamic.

In summary, DOAC represents a tractable co-culture system that facilitates extended interrogation of host–anaerobe interactions. While we use this model here to evaluate reciprocal interactions between *F. nucleatum* and the airway epithelium, this work will undoubtedly benefit future studies focused on anaerobe–host and anaerobe–host–pathogen interactions, and the behavior of facultative anaerobic airway pathogens (e.g., *P. aeruginosa, S. aureus*) under hypoxic microenvironments known to exist *in vivo*. Not only do we anticipate generating a deeper understanding of our microbiota at the epithelial interface under oxygen-limited conditions, we also expect to identify new therapeutic strategies in addition to understanding how existing antimicrobials are impacted by low oxygen. Finally, while we use *F. nucleatum* as our model organism, this work motivates additional studies of the gut, lung, oral cavity, and other sites of infection, where etiological roles of anaerobes have been proposed but specific pathogenic mechanisms remain unclear.

## MATERIALS AND METHODS

### DOAC of epithelial cells

Calu-3 cells were maintained in Eagle’s minimal essential medium (EMEM; Corning, USA) in 10% fetal bovine serum (FBS; Gene) supplemented with 100 U/mL penicillin and 100 µg/mL streptomycin (Gibco) at 37°C in a 5% CO_2_ incubator. Upon reaching 80% confluency, 1 × 10^5^ cells were passaged onto 6.5 mm Transwell culture inserts (24-well, 0.4 µm pore; Corning). When cells reached confluency (~5 days), apical medium was removed to establish an ALI. Polarized cells were maintained for an additional 21–28 days, with basolateral media changes every 48 h to facilitate differentiation and mucus accumulation.

To assemble DOAC, polarized Calu-3 cell-containing Transwell inserts were aseptically mounted in a custom 3D-printed thermoplastic polyurethane gasket sheet (File S1) in a 24-well gas-permeable culture plate (Coy Labs, Grass Lake, MI) containing 800 µL of EMEM per well (Fig. S1). Sterile mineral oil (500 µL) was added to unused wells to prevent gas permeation from the basolateral to apical side of the manifold. Once assembled and covered with a custom-printed sterilized lid (File S2), the apparatus was moved to a culture chamber (Coy) housed in a Coy anaerobic workstation (90% N_2_, 5% H_2_, 5% CO_2_), while mixed blood gas (21% O_2_, 5% CO_2_, 74% N_2_) was delivered (0.2 standard cubic feet per hour) to the base of the plate to oxygenate the basolateral side of the polarized monolayer. We note that the air lock of the anaerobic chamber was not used during transfer to preserve the integrity of the cell cultures.

RPMI 2650 cells were similarly cultured, except Dulbecco’s modified Eagle medium was used as the basal growth medium. Primary nHTBE cells (Lonza Bioscience, CC-2540S) harvested from healthy individuals were expanded using Pneumacult-Ex Plus (Stem Cell Technologies) prior to seeding on 24-well inserts. Cells were maintained at ALI for ~21 days in Pneumacult ALI growth medium (Stem Cell Technologies) at 37°C and 5% CO_2_ in a humidified incubator prior to DOAC.

### Cell culture assays

Calu-3 barrier integrity was determined by TEER measured with a Millicell-ERS2 Volt-Ohm meter (Millipore Sigma). Barrier integrity was further assessed using the Human E-cadherin Quantikine enzyme-linked immunosorbent assay (ELISA) kit (R&D Systems, Minneapolis, MN). Cells were washed with 100 µL of PBS, absorbance was measured at 450 nm using a BioTek Synergy H2 plate reader, and concentrations of E-cadherin were determined against a standard curve according to manufacturer’s instructions. Tight junction formation was assayed using immunofluorescence with an anti-ZO-1 monoclonal antibody (Thermo). To do so, Calu-3 cultures on Transwell inserts were chemically fixed in 4% paraformaldehyde in PBS for 1 h at room temperature. Cells were blocked in 10% goat serum and 1% bovine serum albumin (Sigma) for 15 min. After blocking, cells were incubated with mouse anti-human ZO-1 (AlexaFluor-488 conjugate; ThermoFisher) (5 µg/mL) for 1 h. Membranes were washed, carefully removed from the supporting plastic insert using a biopsy punch, and mounted on glass slides using Vectashield anti-fade mounting medium. Labeled cells were visualized (ex. 480 nm, em. 525 nm) on an Olympus IX83 inverted fluorescence microscope using a 20× objective lens (0.75 numerical aperture). Cytotoxicity was determined by LDH measurements on cell-free supernatants collected from the apical compartment by washing with 100 µL of PBS. LDH was quantified using the Cytotoxicity Detection Kit Plus assay (Roche) according to manufacturer’s instructions. Data were calculated as percent LDH release compared with a lysed control and reported as %LDH release = [(experimental value − low control)/(high control − low control)] × 100.

### Immunoassays

The inflammatory response of Calu-3 cells was measured after 24 h of culture in the anaerobic chamber. To do so, spent medium was collected from each Transwell, and IL-8 and IL-6 were then measured by ELISA per manufacturer instructions (R&D systems). Untreated epithelial cells grown under standard incubator conditions (5% CO_2_) were used as controls.

### Microelectrode measurements

Apical oxygen concentrations were measured using a PreSens Profiling Oxygen Microsensor PM-PSt7 (PreSens, Germany) secured in a motorized automated micromanipulator connected to a Microx 4 portable oxygen meter operated with Profiling Studio 2 software (PreSens). The O_2_ sensor has a tip diameter of 10 µm, spatial resolution of 50 µm, a detection limit of 0.3% O_2_, and high spatial resolution (<50 µm). A two-point sensor calibration was performed by immersing the electrode tip in 100% air-saturated water and an O_2_-free, nitrogen-purged solution of 1% sodium sulfite (Na_2_SO_3_) in 100 mL of water containing 50 µL of a 0.1% cobalt nitrate [Co(NO_3_)_2_] solution in 0.5 mol/L nitric acid. Additional control readings were performed as the electrode moved from ambient air into nitrogen-purged water, and the ambient anaerobic chamber environment (90% _N2_, 5% CO_2_, 5% H_2_) into air-saturated water immediately after transfer to the anaerobic environment. Prior to O_2_ profiling, Transwell depth was determined using digital calipers, and the sensor was positioned at the upper rim using sterile paper as a guide and by visual inspection with assistance from a digital microscope. Vertical O_2_ profiles were collected by setting the micromanipulator to move the sensor through the Transwell along the *z*-axis with the following program: 0–12 mm, 1 mm steps; 12–15 mm, 500 µm; 15–16 mm, 100 µm; 16–17 mm, 10 µm (141 steps total). Measurements were made at 1 s intervals prior to advancement to the next depth. Calu-3 cell and mucus thickness was estimated using previous measurements via confocal microscopy. The estimated insertion depth of the probe tip into the Calu-3 mucus layer was visualized using a digital microscope (Fig. S2). Data were collected by means of the PreSens Profiling Studio 2 software. Measurements were performed on *n* = 3 biological replicates for each condition.

### Bulk RNA sequencing

The transcriptomic response of Calu-3 cells to anoxic culture was determined using RNAseq. Calu-3 cells were cultured at ALI as described above and harvested after 24 and 48 h of (i) normoxic growth under standard incubator conditions, and (ii) DOAC growth without bacterial challenge. At each time point, RNAlater (Invitrogen) was added to the apical and basolateral side of each well. For each condition, RNA was isolated from at least four separate Transwells using the RNeasy Micro Plus kit (Qiagen) according to manufacturer’s instructions. DNase treatment was performed as part of the RNA Clean and Concentrator kit (Zymo). RNA quality (RNA integrity number [RIN] > 9.7) and quantity were assessed using an Agilent Bioanalyzer and RiboGreen, respectively. cDNA libraries were prepared using the SMARTer Universal Low Input RNA Kit (Takara Bio) and submitted for sequencing at the University of Minnesota Genomics Center on the Illumina NovaSeq 6000 platform.

The Ensembl GTF annotation file was filtered to remove annotations for non-protein-coding features. Fastq files were evenly subsampled down to a maximum of 100,000 reads per sample. Data quality in fastq files was assessed with FastQC. Raw reads were mapped to reference Human (Homo_sapiens) genome assembly “GRCh38” using annotation from Ensembl release 98. Gene counts were generated with “featureCounts” of the RSubread package (32). DESeq2 (33) was used to estimate size factors to generate normalized count data, estimate gene-wise dispersions, shrink estimates using type=“ashr,” and perform Wald hypothesis testing (33, 78). Genes with a log_2_ fold change greater than 1 and Benjamini-Hochberg adjusted *P*-value (*P*adj) <0.001 were considered significant.

### Bacterial culture and epithelial challenge

*Fusobacterium nucleatum* subsp. *nucleatum* ATCC 25586 was obtained from Microbiologics (St. Cloud, MN) and was routinely maintained on brain-heart infusion medium supplemented with hemin (0.25 g/L), vitamin K (0.025 g/L), and laked sheep’s blood (5%, vol/vol) (BHI-HKB) in an anaerobic chamber. Forty-eight hours prior to bacterial challenge, Calu-3 cells were incubated in EMEM + FBS without antibiotics. On the day of the challenge, Transwells were assembled in the gas-permeable culture system and transferred into the anaerobic chamber where they were equilibrated for 3 h. Overnight cultures of *F. nucleatum* were grown in BHI. Each individual culture was diluted to a concentration of ~1 × 10^6^ CFU in MEM, and 100 µL of bacterial suspension was added to the apical side of the Calu-3 cells. Co-cultures were then incubated for an additional 24 h. Following the anaerobe challenge, spent medium was collected and analyzed for bacterial cytotoxicity using the LDH colorimetric assay (described above). In a separate experiment, *F. nucleatum* viability was determined using plate enumeration. Briefly, Calu-3 cells were washed three times with 100 µL of PBS and were serially diluted for enumeration on *Fusobacterium* selective agar (Anaerobe Systems).

### SCFA quantification

Short-chain fatty acid concentrations were determined using gas chromatography mass spectrometry. Briefly, apical media from treated and untreated Calu-3 cultures was collected and pooled from triplicate wells (~300 µL total) and filter sterilized using micro-centrifugal filters (Thermo Scientific, F2517-7) (*n* = 4 for each condition). Filtrate was stored at −80°C prior to shipment to Creative Proteomics (Shirley, NY). There, free short-chain fatty acids were derivatized using methyl chloroformate in 1-propanol yielding propyl esters before subsequent liquid–liquid extraction into hexane and analysis on an SLB-5ms (30 × 0.25 × 1.0 μm) column and detection using gas chromatography-electron ionization-mass spectrometry (GC-EI-MS) in positive selected ion mobility (SIM) mode. Deuterium-labeled acetate, propionate, butyrate, isobutyrate, valerate, isovalerate, and hexanoic acid were used as internal standards. Only acetate, propionate, and butyrate were quantifiable. All other metabolites fell below the limit of detection.

### Hybridization chain reaction

To visualize *F. nucleatum* colonization of Calu-3 cells, we used HCR-FISH. Briefly, DOAC co-cultures were fixed in 2% anaerobic paraformaldehyde for 4 h, and Transwells were carefully removed from their plastic casing using a dermal biopsy punch. Membranes were deposited on glass slides and subjected to HCR using previously published protocols (27).

#### Hybridization

Membranes were pre-treated with 200 µL of HCR hybridization buffer (Molecular Instruments, Eagle Rock, CA) for 10 min at 37°C followed by 100 µL of hybridization solution containing 0.4 pmol of a custom-designed *F. nucleatum* probe set (Molecular Instruments). Slides were incubated for another 12 h at 37°C, followed by a rinse in a gradient (100%, 75%, 50%, 25%, 0%) of wash buffer in 5× sodium chloride sodium citrate Tween (SSCT) buffer for 15 min each.

#### Amplification

After washing, 200 µL of HCR amplification buffer (Molecular Instruments) was applied to each slide and incubated at room temperature. Concurrently, AlexaFluor-647-conjugated DNA hairpin pairs (2 µL of a 3 µM stock) were heated to 95°C for 90 s in separate PCR tubes then cooled to room temperature. One hundred microliters of amplification buffer containing the hairpin pair was applied to each sample and incubated overnight. Slides were carefully rinsed in 5× SSCT (2 × 30 min, 1 × 5 min).

#### Microscopy

Fifty microliters of Diamond anti-fade mounting reagent was added to each slide and sealed with a coverslip. Samples were imaged on an Olympus IX83 inverted fluorescent microscope with a transmitted Koehler illuminator using 20× (Calu-3) and 63× oil (*F. nucleatum*) objective lenses. Calu-3 nuclei and HCR probes were visualized using excitation/emission wavelengths of 377/447 nm (4',6-diamidino-2-phenylindole, DAPI) and 628/692 nm (Cy5), respectively. Images were captured on a Hamamatsu ORCA-Flash4.0 V2 digital CMOS camera, and post-acquisition image processing was performed using CellSens software (v.1.14, Olympus). Uninfected control samples and co-cultures incubated without RNA probes (i.e., fluorescent hairpins only) were also evaluated.

### *F. nucleatum* RNA analysis

After Calu-3 co-culture with *F. nucleatum,* 250 µL of TRIzol (ThermoFisher) was added to each Transwell, bacteria and epithelial cells were both gently pipetted off the surface, and samples were pooled in quadruplicate to increase RNA yields. Planktonic cultures of *F. nucleatum* were grown in parallel in EMEM for 24 h, pelleted by centrifugation, and resuspended in 750 µL prior to RNA extraction. After 5 min of TRIzol incubation, 200 µL of chloroform was added, tubes were agitated by hand, and centrifuged at 12,000 rpm for 15 min at 4°C. The aqueous phase was removed and mixed with an equal volume of 95% ethanol. The mixture was then cleaned up using the RNA Clean & Concentrator-5 kit (Zymo) including the on-column DNase I treatment according to the manufacturer’s protocol. RNA concentration was determined using the Qubit Broad Range RNA kit prior to storage at −80°C. RNA libraries were prepared and sequenced at SeqCoast Genomics (Portsmouth, NH). Briefly, samples were prepared for sequencing using an Illumina Stranded Total RNA Prep Ligation with Ribo-Zero Plus Microbiome and unique dual indexes. Sequencing was performed on the Illumina NextSeq 2000 platform using a 300 cycle flow cell kit to produce 2 × 150 bp reads. Read demultiplexing, read trimming, and run analytics were performed using DRAGEN v.3.10.12 software. Raw fastq files were aligned to the *F. nucleatum* subsp. *nucleatum* 25586 genome (NCBI RefSeq NZ_CP028101.1) using the Subread aligner, implemented using RSubread. Gene counting was performed using both “featureCounts” and RSubread. DESeq2 was used to estimate size factors and carry out variance-stabilizing transformation and statistical testing via the Wald test. Genes with a log_2_ fold change greater than 1 with a two-tailed Benjamini-Hochberg adjusted *P*-value (*P*adj) <0.001 were considered significant.

### Single-cell RNA sequencing and analysis

nHTBE cells grown on Transwell inserts were incubated in antibiotic-free Pneumocult medium for 48 h prior to bacterial challenge. nHTBEs were assembled in the DOAC system and transferred into the anaerobic chamber. After 3 h of nHTBE equilibration, an overnight culture of *F. nucleatum* grown in BHI was diluted to a concentration of 1 × 10^6^ CFU in Pneumacult medium, and 10 µL of bacterial suspension was added to the apical side of the nHTBE cells in the anaerobic chamber. Co-cultures were incubated for 24 h prior to epithelial cell homogenization and cell capture.

Samples were prepared for scRNAseq as described previously (46). Briefly, single-cell suspensions were washed with PBS + 5% FBS, resuspended in 200 µL of PBS + 5% FBS, passed through a 70 µm cell strainer, and placed on ice. Cells were counted using trypan blue on a BioRad TC20 automated cell counter. A targeted cell input of 5,000 cells per condition was used to generate gel bead-in emulsion (GEMs). The Chromium Next GEM Single Cell 3´ Gel beads v.3.1 kit (10X Genomics, Pleasanton, CA) was used to create GEMs following manufacturer’s instructions. Captured GEMS were used for cDNA synthesis and library preparation using the Chromium Single Cell 3´ Library Kit v.3.1, followed by sequencing using the Illumina NovaSeq platform.

Raw count matrices were generated with Cell Ranger (v.3.0.1) for alignment to the human reference genome (Homo_sapiens.GRCh38). The resulting raw count matrix for each experimental data set was imported into an R pipeline using Seurat (v.4.1.0) (79) for quality control, normalization, integration, clustering, and differential expression analysis. Data were filtered to remove low-quality cells using the following criteria: number of unique molecular identifiers (UMI counts) per cell ≥500, number of genes per cell ≥250, number of genes detected per UMI ≥0.8, mitochondrial ratio less than 0.25, and genes expressed in ≥10 cells. “SCTransform” was used to normalize the data and regress out variation due to mitochondrial gene expression. Cells were integrated across conditions using default parameters except all 300 most variable genes identified by SCTransform were used. Cell clustering was performed based on the first 20 principal components using the FindClusters function with a 0.4 resolution. Conserved markers in each cluster were identified using FindConservedMarkers. Cell types for each cluster were then identified using the top 10 markers by average log fold change across groups. This list of top markers was compared to known cell markers for basal cells, secretory cells, ciliated cells, and ionocytes (43). Clusters with a minimum of 2 out of the 10 top markers corresponding to a particular cell type were labeled as such. DESeq2 (v.1.34.0) was then used to perform pseudobulk differential expression analysis across the different cell types accounting for *F. nucleatum* challenge. Specifically, this package was used to estimate size factors, normalized count data, estimate gene-wise dispersions, shrink estimates using type=“apeglm” (80), and perform Wald hypothesis testing. Genes with a log_2_ fold change greater than 1 and Benjamini-Hochberg adjusted *P*-value <0.05 were considered significant. Pattern recognition receptor gene expression was visualized in the untreated population using Nebulosa (v.1.4.0) plot_density function. All code and data files are shared at https://github.com/Hunter-Lab-UMN/Moore_PJ_2022.

## Data Availability

Code and data files are shared at https://github.com/Hunter-Lab-UMN/Moore_PJ_2022 and NCBI Gene Expression Omnibus (GEO) accession no. GSE218124 (https://ncbi.nlm.nih.gov/bioproject/PRJNA902376).

## SUPPLEMENTAL MATERIAL

Data set S1 mbio.01338-24-s0001.csv DOAC 24-h RNASeq.

Data set S2 mbio.01338-24-s0002.csv DOAC 48-h RNASeq.

Data set S3 mbio.01338-24-s0003.csv *F. nucleatum* RNAseq.

Data set S4 mbio.01338-24-s0004.xlsx Single-cell RNASeq data.

File S1 mbio.01338-24-s0005.txt SolidWorks print file for DOAC lid.

File S2 mbio.01338-24-s0006.txt SolidWorks print file for DOAC gasket.

Supplemental material mbio.01338-24-s0007.pdf Figures S1-S8; Table S1.

ASM does not own the copyrights to Supplemental Material that may be linked to, or accessed through, an article. The authors have granted ASM a non-exclusive, world-wide license to publish the Supplemental Material files. Please contact the corresponding author directly for reuse.
