# Supplementary material for: Dual oxic-anoxic co-culture enables direct study of anaerobe–host interactions at the airway epithelial interface
Source: mBio. 2025 Apr 9;16(5):e01338-24. doi: 10.1128/mbio.01338-24 (PMC12077211; doi:10.1128/mbio.01338-24)
Supplement: Supplemental material — Figures S1-S8; Table S1. [file mbio.01338-24-s0007.pdf]

Supplemental Data

Dual oxic-anoxic co-culture enables direct study of anaerobe-host interactions at the airway epithelial interface

P.J. Moore et al.

Document S1. Figures S1-S8, Table S1

Data S1. Excel file containing RNAseq data from DOAC at 24h

Data S2. Excel file containing RNAseq data from DOAC at 48h

Data S3. Excel file containing RNAseq data from *F. nucleatum* after 24h of co-culture

Data S4. Excel file containing single-cell RNA sequencing data.

File S1. SolidWorks print file for DOAC gasket

File S2. SolidWorks print file for DOAC lid

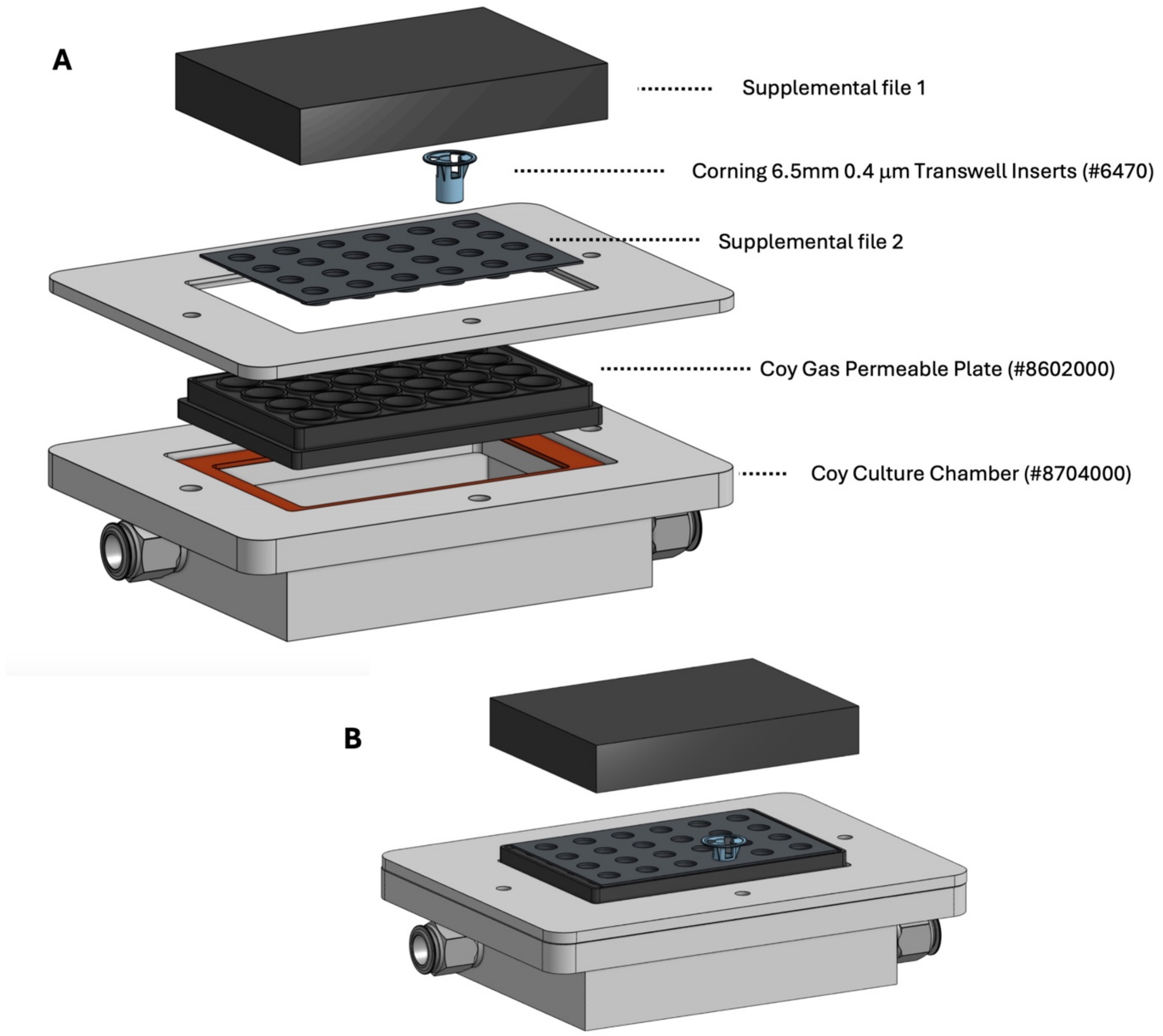

Figure S1. Schematic of an **(A)** unassembled and **(B)** assembled DOAC platform. Transwells are mounted in a custom 3D-printed thermoplastic polyurethane gasket mounted on a gas permeable multi-well plate. Blood gas is delivered and removed through cable glands mounted to the basolateral compartment of the Transwell-containing apparatus.

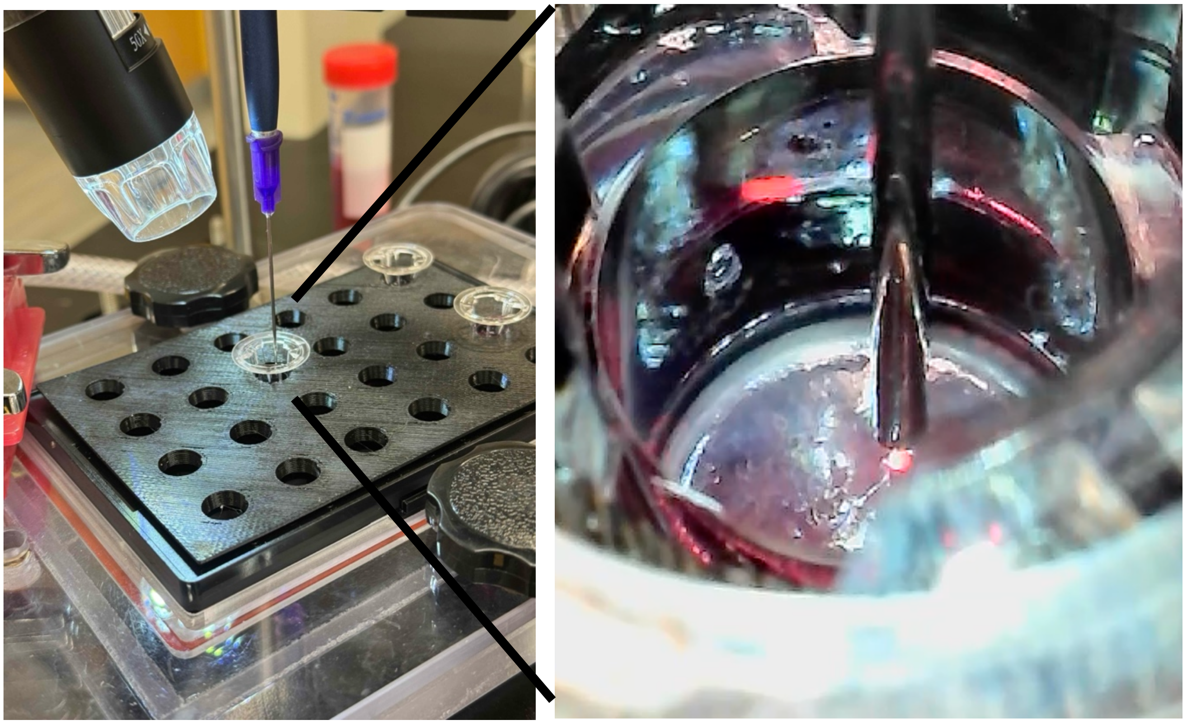

Figure S2. Oxygen microprofiling. Apical oxygen concentrations were determined using a fiber-optic oxygen microsensor mounted on an automated micromanipulator connected to a Microx4 portable oxygen meter operated with Profiling Studio 2 software (PreSens). Microsensor placement and depth was guided with the assistance of a digital microscope.

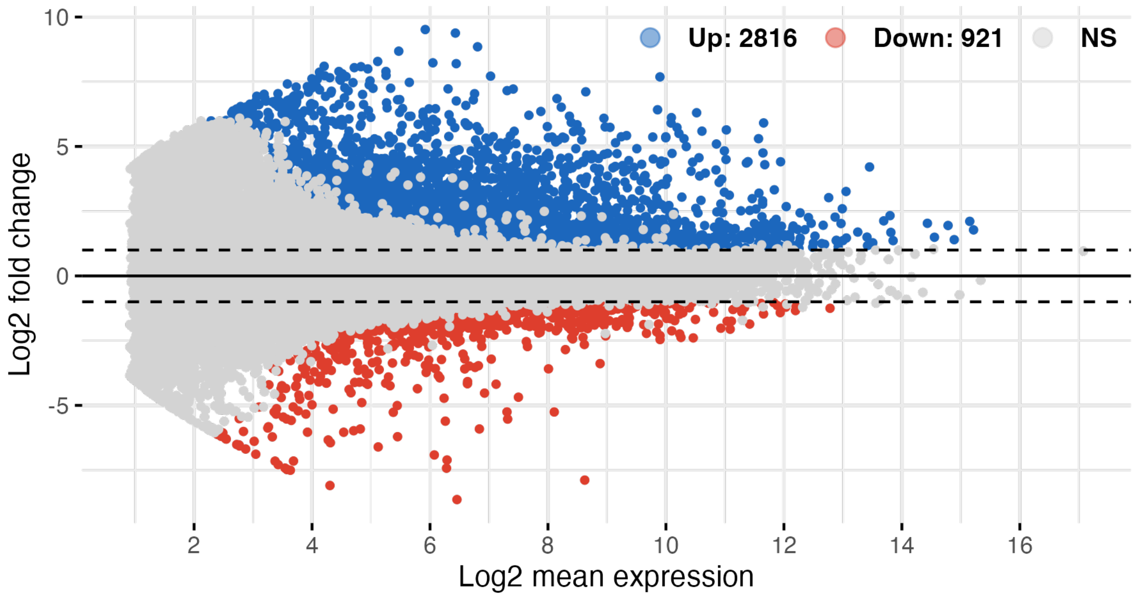

Figure S3. MA plot representation of Calu-3 gene expression under DOAC conditions after 48h relative to normoxic culture at ALI (2816 genes upregulated, 921 downregulated; log2fc >1, padj (0.001).

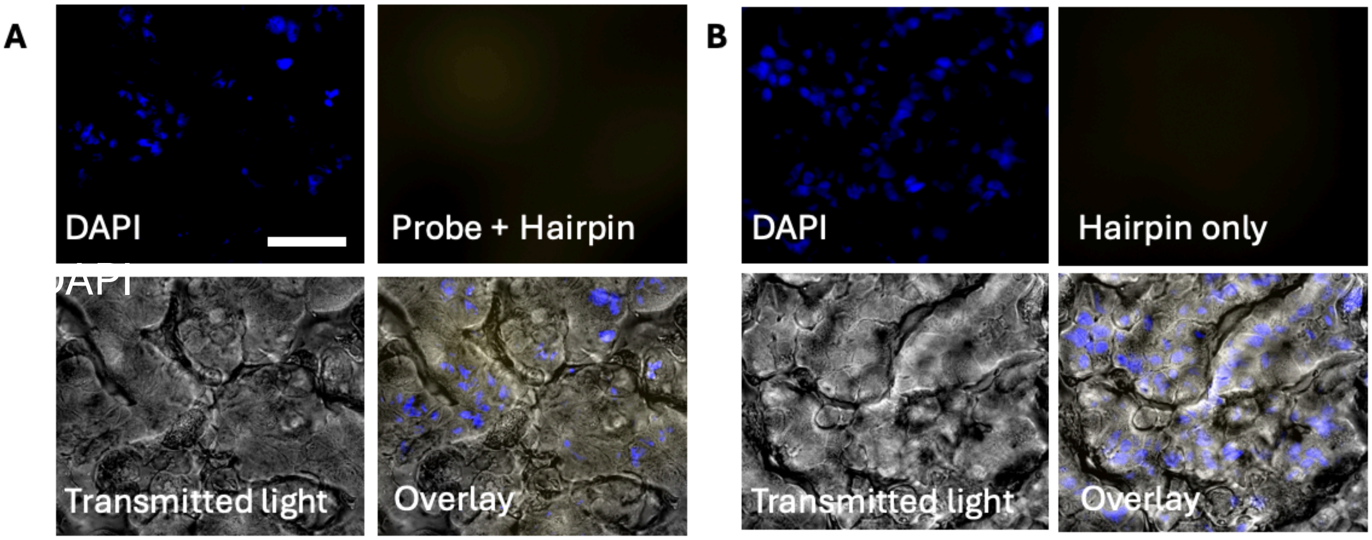

Figure S4. Hybridization chain reaction (HCR) imaging validation. (A) Fixed, uninfected Calu-3 cells treated exhibited negligible background fluorescence when treated with *F. nucleatum* 16S rRNA probes and fluorescent hairpins. (B) Infected Calu-3 cells exhibited no fluorescent signal when treated with hairpins only (i.e. no 16S probes). Together, these data confirm the specificity of HCR probes for *F. nucleatum*. Bar = 20μm.

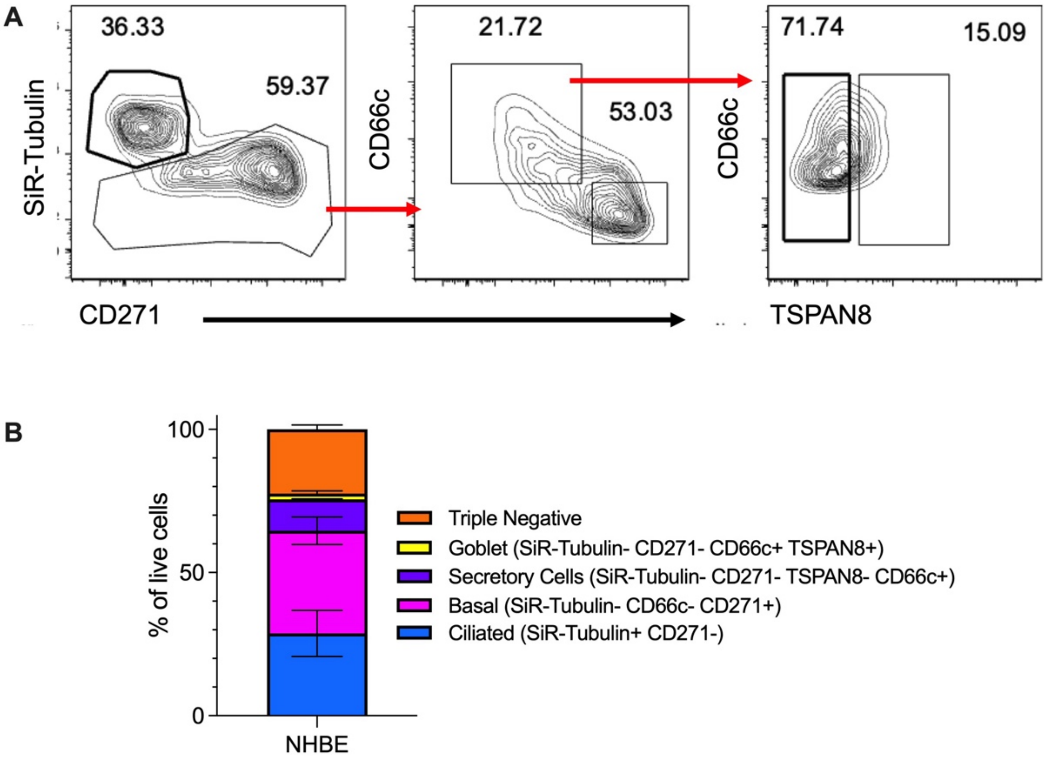

Figure S5. (A) Gating strategy for identifying epithelial cell types in NHBE cell cultures (gated from live, singlet, Live/Dead- cells). Data depicts NHBE cells analyzed three weeks post-ALI. **(B)** Stacked bar plot of average cell composition from three individual wells. Error bars indicate the SEM. Flow cytometry was performed as described previously (Roach et al., 2024).

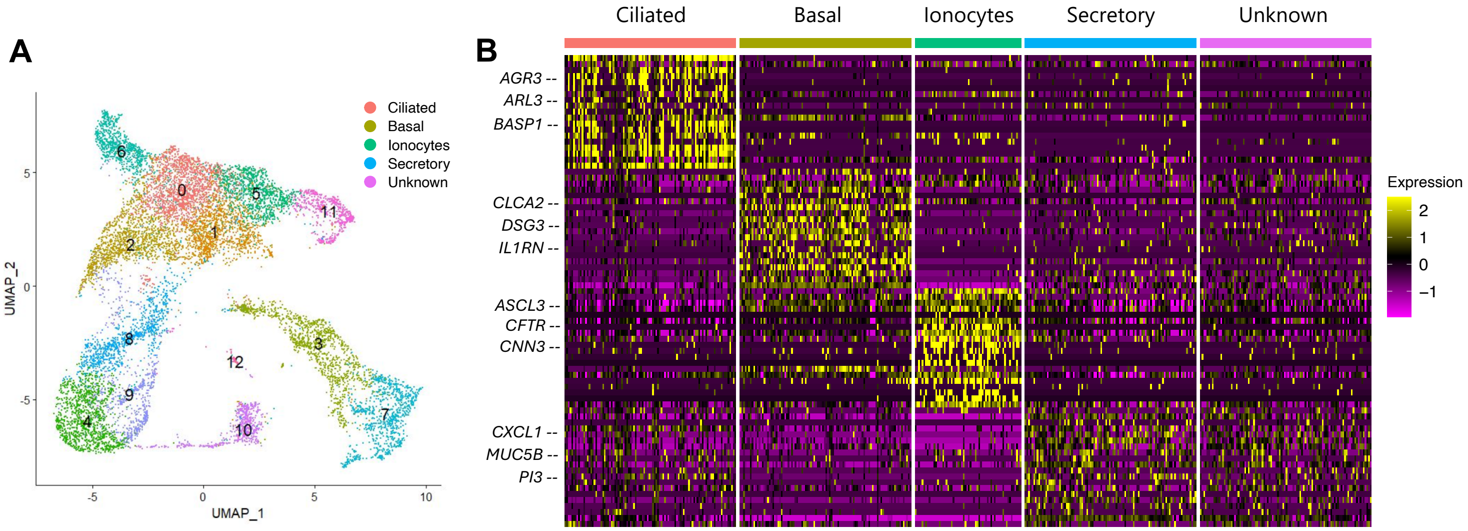

Figure S6. (A) UMAP projection of nHTBEs showing 4 major cell types (and unknown cells) from 13 distinct clusters. (B) Scaled expression of the top differentially expressed genes between cell clusters that informed assignment of cell type identity to specific subsets.

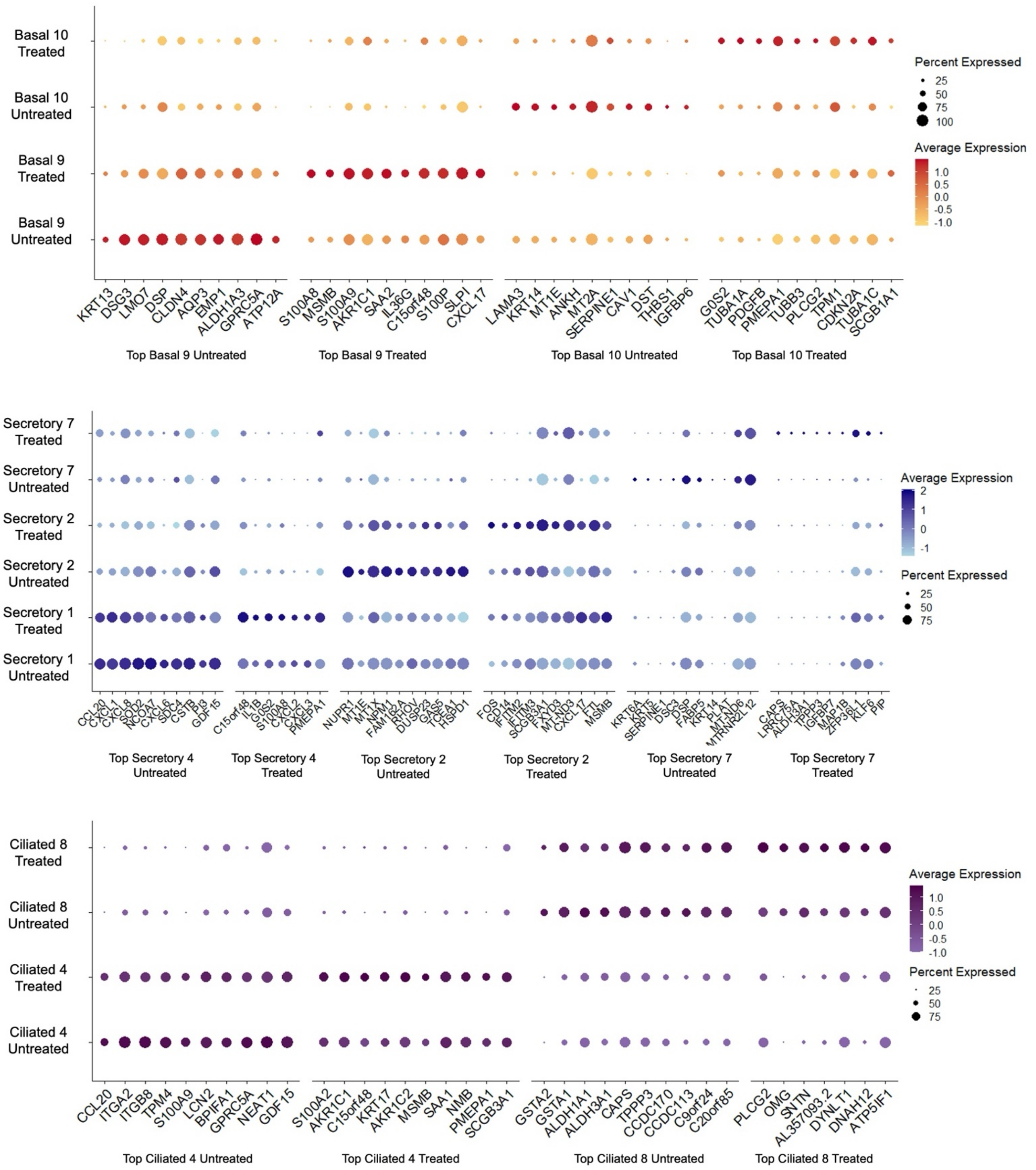

Figure S7. Dot plot representation of differential gene expression between cell type sub-clusters (see Fig.5C). Shown are the top 7-10 most highly expressed genes in each cluster, compared to their relative expression in the other clusters. Dot size denotes the percentage of cells in a cluster expressing a given gene while dot density denotes average expression across each cell (i.e. the biggest and darkest circle is the most highly expressed in the most cells, and vice versa).

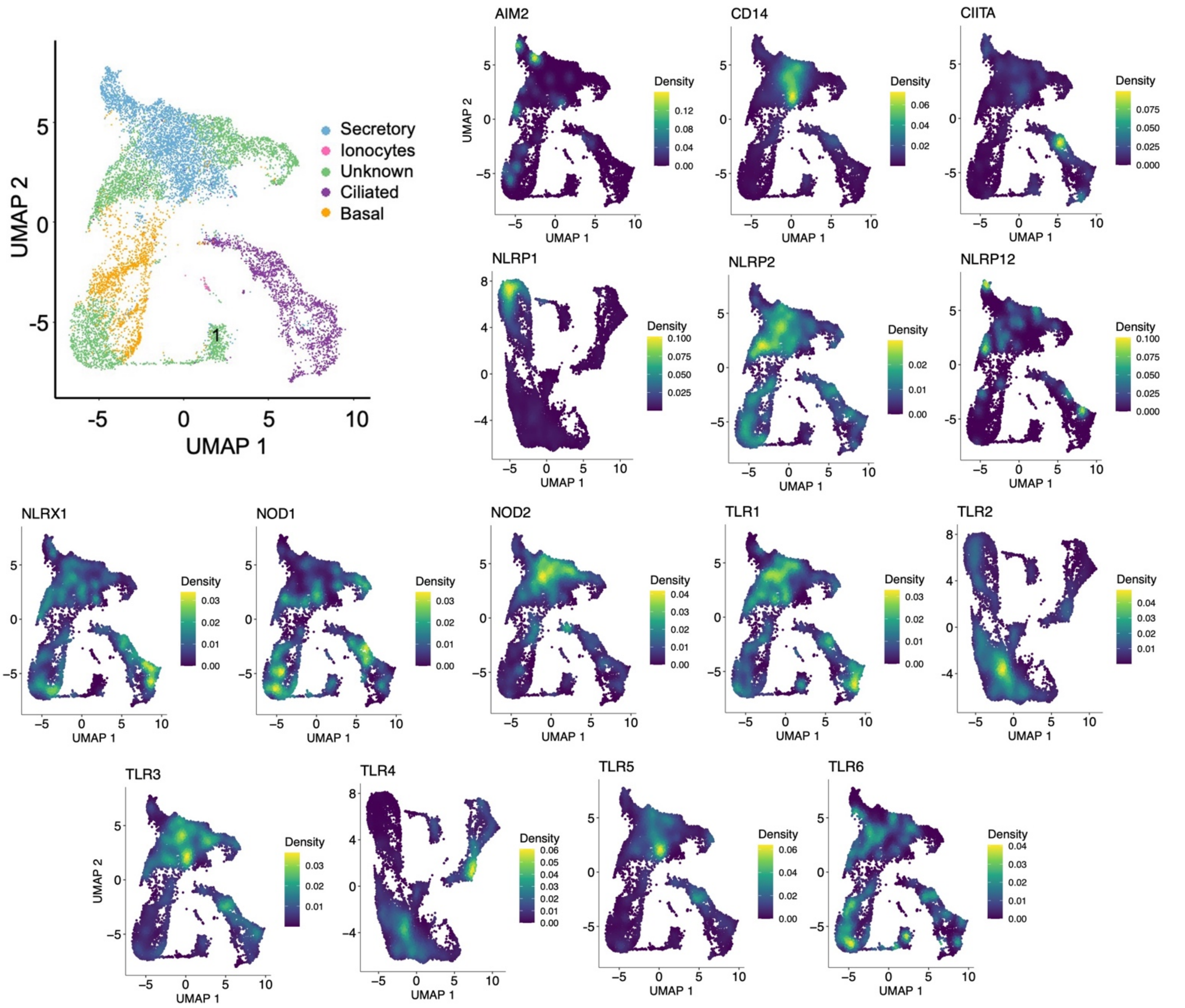

Figure S8. Heterogeneous expression of pattern recognition receptors by epithelial cell type. (Top left) UMAP projection of nHTBEs clustered based on global gene expression patterns. The cell identity of each cell cluster was determined based on known marker genes. All other Nebulosa plots reveal the cell-type specific expression patterns of Toll-like receptors (TLRs), nucleotide-binding oligomerization domain (NOD) receptors, NOD-Leucine Rich Repeat-containing receptors (NLR), and other pattern recognition receptors (PRRs) on nHTBEs.

Table S1. Differential gene expression analysis of *F. nucleatum* during epithelial colonization versus planktonic culture. Genes are ordered by log2 fold change. Shown are genes with a log2 fold change >1, padj<0.001. The full dataset can be found in Data S3.

locus_tag	FN_tag	gene	function	KEGG	baseMean	log2fc	lfcSE	stat	pvalue	padj
C7Y58_03075	FN1957		hypothetical protein	Hypothetical	5474.2330	6.142733	0.695875	7.3903	1.46E-13	7.92E-12
C7Y58_03405	FN1886		hypothetical protein	Hypothetical	16495.445	5.942710	0.649955	7.6046	2.86E-14	1.73E-12
C7Y58_03410	FN1885		hemolysin III	Virulence	22236.620	5.848706	0.590308	8.2138	2.14E-16	1.63E-14
C7Y58_03080	FN1956		hypothetical protein	Hypothetical	1560.4230	5.705017	0.680336	6.9157	4.65E-12	1.80E-10
C7Y58_01630	FN0120		hypothetical protein	Hypothetical	37.263827	5.131459	1.029872	4.0116	6.03E-05	7.12E-04
C7Y58_00470	FN1590		lipoprotein	Hypothetical	10713.138	5.109532	0.844337	4.8671	1.13E-06	1.95E-05
C7Y58_06160	FN0622		N-glycosylase/DNA lyase	DNA Replication	5985.1927	4.944087	0.738207	5.3427	9.15E-08	2.00E-06
C7Y58_06340	FN0658	*metQ*	ABC transporter substrate-binding protein	Transport	6421.8460	4.596500	0.549327	6.5470	5.87E-11	2.01E-09
C7Y58_09175	FN1234		CAP domain-containing protein	Hypothetical	3177.1996	4.522951	0.652931	5.3955	6.83E-08	1.52E-06
C7Y58_09585	FN1315		hypothetical protein	Hypothetical	1955.1969	4.482795	0.382208	9.1122	8.07E-20	1.04E-17
C7Y58_06345	FN0659	*metI*	ABC transporter permease	Transport	2385.5218	4.470615	0.699073	4.9645	6.88E-07	1.29E-05
C7Y58_09055	FN1209	*yhbY*	ribosome assembly RNA-binding protein YhbY	Translation	177.39957	4.370127	0.463464	7.2715	3.55E-13	1.78E-11
C7Y58_06830	FN0757	*scpB*	SMC-Scp complex subunit ScpB	DNA Replication	2054.3184	4.208682	0.363157	8.8355	9.96E-19	1.08E-16
C7Y58_06350	FN0660	*metN*	methionine import ATP-binding protein MetN	Transport	1755.4898	4.201448	0.719379	4.4502	8.58E-06	1.27E-04
C7Y58_06165	FN0623	*mleP*	AEC family transporter (Malonate)	Transport	10986.898	4.155164	0.799640	3.9457	7.96E-05	9.08E-04
C7Y58_08490	FN1093		Rrf2 family transcriptional regulator	Signaling	5640.8017	4.128894	0.578941	5.4045	6.50E-08	1.47E-06
C7Y58_09040	FN1206	*tlyA*	TlyA family rRNA (cytidine-2'-O)-methyltransferase	Translation	5364.9578	4.071002	0.267303	11.488	1.50E-30	4.41E-28
C7Y58_09050	FN1208	*dxs*	1-deoxy-D-xylulose-5-phosphate synthase	Metabolism	15886.615	4.022773	0.218420	13.839	1.48E-43	3.04E-40
C7Y58_06835	FN0758	*mreB*	rod shape-determining protein	Ultrastructure	22040.348	4.014432	0.229671	13.124	2.37E-39	2.43E-36
C7Y58_09060	FN1210	*rnj*	ribonuclease J	Translation	29979.848	4.011229	0.304463	9.8902	4.59E-23	7.25E-21
C7Y58_09045	FN1207		HD domain-containing protein	Hypothetical	11848.285	3.969437	0.288688	10.285	8.15E-25	1.67E-22
C7Y58_08485	FN1092		DUF1385 domain-containing protein	Hypothetical	34675.604	3.966744	0.646354	4.5899	4.43E-06	7.06E-05
C7Y58_04895	FN0369	*rimI*	ribosomal-protein-alanine N-acetyltransferase	Translation	695.31123	3.941054	0.323095	9.1027	8.81E-20	1.06E-17
C7Y58_09590	FN1316		Nif3-like dinuclear metal center hexameric protein	Transcription	2617.0033	3.892283	0.283181	10.213	1.72E-24	3.22E-22
C7Y58_05470	FN0480		hypothetical protein	Hypothetical	9355.2558	3.861316	0.321929	8.8880	6.22E-19	7.10E-17
C7Y58_05465	FN0479	*rpoE*	RNA polymerase sigma factor	Transcription	3226.0376	3.847064	0.542256	5.2504	1.52E-07	3.18E-06
C7Y58_06825	FN0756	*rluB*	rRNA pseudouridine synthase	Translation	6840.7835	3.842014	0.478620	5.9379	2.89E-09	8.01E-08
C7Y58_06820	FN0755	*gatC*	Asp-tRNA(Asn)/Glu-tRNA(Gln) amidotransferase	Translation	2138.8860	3.839101	0.417483	6.8005	1.04E-11	3.89E-10
C7Y58_01225	FN1745	*metB*	cystathionine beta-lyase	Metabolism	1781.3210	3.724050	0.390414	6.9773	3.01E-12	1.24E-10
C7Y58_04885	FN0367		hypothetical protein	Hypothetical	1328.4898	3.686737	0.383444	7.0068	2.44E-12	1.07E-10
C7Y58_05475	FN0481		hypothetical protein	Hypothetical	12049.709	3.624004	0.303404	8.6485	5.22E-18	4.66E-16
C7Y58_01090	FN1717	*ligA*	DNA ligase (NAD(+)) LigA	DNA Replication	2745.7877	3.598962	0.402476	6.4574	1.06E-10	3.47E-09
C7Y58_01815	FN0082	*eutM*	ethanolamine utilization protein EutM	Metabolism	175.77463	3.581423	0.371233	6.9536	3.56E-12	1.43E-10
C7Y58_04500	FN0289		hypothetical protein	Hypothetical	133.00803	3.576176	0.395557	6.5127	7.38E-11	2.44E-09
C7Y58_05970	FN0583		peptidase	Virulence	378.03964	3.527671	0.576529	4.3842	1.16E-05	1.68E-04
C7Y58_09025	FN1203	*rbgA*	ribosome biogenesis GTPase YlqF	Translation	1342.2708	3.523280	0.287932	8.7634	1.89E-18	1.85E-16
C7Y58_04890	FN0368	*purB*	adenylosuccinate lyase	Metabolism	19442.122	3.520426	0.315444	7.9900	1.35E-15	9.23E-14
C7Y58_01835	FN0078	*eutA*	ethanolamine ammonia-lyase reactivating factor EutA	Metabolism	1556.7419	3.510244	0.401972	6.2448	4.24E-10	1.32E-08
C7Y58_01830	FN0079	*eutB*	ethanolamine ammonia lyase large subunit	Metabolism	7871.7143	3.509049	0.361673	6.9373	4.00E-12	1.58E-10
C7Y58_05980	FN0585	*czcR*	DNA-binding response regulator	Signaling	1354.6753	3.508760	0.419702	5.9774	2.27E-09	6.47E-08
C7Y58_09845	FN1368		amidophosphoribosyltransferase	Metabolism	199.33415	3.481594	0.343845	7.2171	5.31E-13	2.60E-11
C7Y58_05975	FN0584		hypothetical protein	Hypothetical	1158.8992	3.465726	0.590215	4.1776	2.95E-05	3.83E-04
C7Y58_01825	FN0080	*eutC*	ethanolamine ammonia-lyase light chain	Metabolism	5010.5944	3.414488	0.315945	7.6421	2.14E-14	1.33E-12
C7Y58_01820	FN0081	*eutL*	ethanolamine utilization microcompartment protein	Metabolism	1405.1060	3.391285	0.336718	7.1017	1.23E-12	5.75E-11
C7Y58_04880	FN0366	*glmM*	phosphoglucosamine mutase	Metabolism	22071.944	3.374390	0.202595	11.719	1.01E-31	3.45E-29
C7Y58_09035	FN1205	*sppA*	S41 family peptidase	Metabolism	4131.9559	3.323894	0.229627	10.120	4.49E-24	7.69E-22
C7Y58_04855	FN0361	*ATPF*	ATP synthase F1 subunit delta	Metabolism	932.67171	3.301181	0.316185	7.2779	3.39E-13	1.74E-11
C7Y58_04865	FN0363	*ATPF*	ATP synthase subunit C	Metabolism	974.34551	3.292849	0.251417	9.1196	7.54E-20	1.03E-17
C7Y58_05965	FN0582	*lolD*	lipoprotein-releasing system ATP-binding protein LolD	Transport	1623.2422	3.249659	0.335249	6.7104	1.94E-11	6.99E-10
C7Y58_04875	FN0365	*atpI*	hypothetical protein	Metabolism	4574.7257	3.208685	0.251254	8.7906	1.49E-18	1.53E-16
C7Y58_08010	FN0998		diguanylate phosphodiesterase	Signaling	11359.829	3.163409	0.410981	5.2640	1.41E-07	2.98E-06
C7Y58_09030	FN1204	*rsmI*	16S rRNA (cytidine(1402)-2'-O)-methyltransferase	Translation	12528.860	3.157688	0.258698	8.3405	7.40E-17	5.93E-15
C7Y58_08015	FN0999		peptidase M42	Metabolism	6005.6709	3.114861	0.308684	6.8512	7.32E-12	2.79E-10
C7Y58_08955	FN1188		hypothetical protein	Hypothetical	4240.5855	3.071989	0.380581	5.4442	5.20E-08	1.26E-06
C7Y58_05960	FN0581	*lolE*	ABC transporter permease	Transport	3816.8059	3.060762	0.479543	4.2973	1.73E-05	2.34E-04
C7Y58_09835	FN1366	*tpi*	triose-phosphate isomerase	Metabolism	1209.8398	3.055443	0.352527	5.8305	5.52E-09	1.51E-07
C7Y58_01220	FN1744		transporter	Transport	485.37615	3.054077	0.493015	4.1663	3.10E-05	3.97E-04
C7Y58_04870	FN0364	*ATPF*	ATP synthase F0 subunit A	Metabolism	7195.2659	3.052545	0.272409	7.5347	4.89E-14	2.79E-12
C7Y58_06815	FN0754	*gatA*	Asp-tRNA(Asn)/Glu-tRNA(Gln) amidotransferase	Translation	22064.075	3.034869	0.351661	5.7864	7.19E-09	1.94E-07
C7Y58_04900	FN0370	*lepB*	signal peptidase I	Transport	3372.4831	3.026179	0.231991	8.7338	2.46E-18	2.30E-16
C7Y58_09840	FN1367		methyl-accepting chemotaxis protein	Virulence	686.24563	3.017192	0.392069	5.1449	2.68E-07	5.35E-06
C7Y58_03670	FN1834	*exbB*	MotA/TolQ/ExbB proton channel family protein	Transport	3339.4108	2.992942	0.345628	5.7661	8.11E-09	2.16E-07
C7Y58_04860	FN0362	*ATPF*	ATP synthase subunit B	Metabolism	10140.389	2.968941	0.264253	7.4509	9.27E-14	5.14E-12
C7Y58_07430	FN0881	*evrC*	multidrug ABC transporter permease (Viologen	Transport	237.39616	2.966086	0.244375	8.0453	8.60E-16	6.09E-14
C7Y58_01810	FN0083	*eutM*	ethanolamine utilization microcompartment protein	Metabolism	1027.1815	2.945475	0.394478	4.9317	8.15E-07	1.49E-05
C7Y58_03695	FN1829		hypothetical protein	Hypothetical	945.38163	2.895972	0.432920	4.3794	1.19E-05	1.70E-04
C7Y58_03665	FN1835		hypothetical protein	Hypothetical	1310.1124	2.891182	0.417747	4.5270	5.98E-06	9.38E-05
C7Y58_03355	FN1899		lipoprotein	Virulence	11899.788	2.869142	0.418108	4.4704	7.80E-06	1.17E-04
C7Y58_03680	FN1832	*tonB*	energy transducer TonB	Transport	5020.8247	2.853810	0.418859	4.4258	9.61E-06	1.40E-04
C7Y58_07230	FN0841		hypothetical protein	Hypothetical	3381.2691	2.850705	0.375524	4.9283	8.29E-07	1.50E-05
C7Y58_08960	FN1189		hypothetical protein	Hypothetical	3021.6235	2.830153	0.417881	4.3796	1.19E-05	1.70E-04
C7Y58_09020	FN1202	*nadE*	NAD(+) synthetase	Metabolism	5258.4471	2.812596	0.231915	7.8157	5.46E-15	3.51E-13
C7Y58_07235	FN0842		hypothetical protein	Hypothetical	4382.7223	2.791561	0.450775	3.9743	7.06E-05	8.19E-04
C7Y58_06520	FN0674		EamA/RhaT family transporter	Transport	9129.8698	2.775707	0.285806	6.2129	5.20E-10	1.57E-08
C7Y58_06525	FN0675	*groEL*	chaperonin GroL	Translation	4354.4017	2.746316	0.248337	7.0320	2.04E-12	9.29E-11
C7Y58_03675	FN1833	*exbD*	biopolymer transporter ExbD	Transport	5134.479	2.706332	0.340241	5.0150	5.30E-07	9.99E-06
C7Y58_06315	FN0653		hypothetical protein	Hypothetical	6120.9546	2.702114	0.312979	5.4384	5.38E-08	1.28E-06
C7Y58_05550	FN0497		type II toxin-antitoxin system RelE/ParE family toxin	Virulence	1101.9739	2.685035	0.394534	4.2709	1.95E-05	0.000261
C7Y58_06810	FN0753	*gatB*	Asp-tRNA(Asn)/Glu-tRNA(Gln) amidotransferase	Translation	8850.8944	2.683446	0.367301	4.5832	4.58E-06	7.23E-05
C7Y58_04850	FN0360	*ATPF*	ATP synthase subunit alpha	Metabolism	29562.503	2.653552	0.274996	6.0129	1.82E-09	5.34E-08
C7Y58_09830	FN1365	*ychF*	redox-regulated ATPase YchF	Translation	7161.1330	2.599286	0.219181	7.2966	2.95E-13	1.55E-11
C7Y58_05985	FN0586		sensor histidine kinase	Signaling	2949.7448	2.588244	0.382268	4.1547	3.26E-05	4.15E-04
C7Y58_06320	FN0654	*pgk*	phosphoglycerate kinase	Metabolism	14201.273	2.563565	0.223972	6.9810	2.93E-12	1.23E-10
C7Y58_06530	FN0676	*groES*	co-chaperone GroES	Translation	199.14338	2.553995	0.304478	5.1037	3.33E-07	6.58E-06
C7Y58_09995	FN1399		hypothetical protein	Hypothetical	194.92909	2.537034	0.381489	4.0290	5.60E-05	6.73E-04
C7Y58_04845	FN0359	*ATPF*	ATP synthase subunit gamma	Metabolism	10003.877	2.532694	0.286244	5.3544	8.58E-08	1.89E-06
C7Y58_01805	FN0084		acetaldehyde dehydrogenase (acetylating)	Metabolism	3067.6490	2.530564	0.380982	4.0174	5.88E-05	7.01E-04

C7Y58_04840	FN0358	*ATPF*	ATP synthase subunit beta	Metabolism	19697.803	2.521402	0.211185	7.2041	5.84E-13	2.79E-11
C7Y58_05870	FN0562	*sepF*	cell division protein SepF	Chromosome	1338.5511	2.516955	0.352731	4.3005	1.70E-05	2.32E-04
C7Y58_03705	NA			Hypothetical	2915.5570	2.511025	0.354942	4.2570	2.07E-05	2.73E-04
C7Y58_02970	FN1978	*ftsH*	ATP-dependent zinc metalloprotease FtsH	Metabolism	18626.881	2.505740	0.374878	4.0166	5.90E-05	7.01E-04
C7Y58_03290	FN1912	*ubiD*	UbiD family decarboxylase	Metabolism	7608.9386	2.480864	0.211087	7.0154	2.29E-12	1.02E-10
C7Y58_04830	FN0356	*GLO1*	lactoylglutathione lyase	Metabolism	2368.6194	2.470299	0.194785	7.5483	4.41E-14	2.59E-12
C7Y58_04835	FN0357		ATP synthase epsilon chain	Metabolism	3302.0216	2.442619	0.240658	5.9944	2.04E-09	5.91E-08
C7Y58_01775	FN0089	*eutH*	ethanolamine utilization protein EutH	Metabolism	1500.4884	2.371679	0.347588	3.9462	7.94E-05	9.08E-04
C7Y58_05545	FN0496		CopG family transcriptional regulator	Signaling	409.49363	2.341149	0.215428	6.2255	4.80E-10	1.47E-08
C7Y58_01100	FN1719		hypothetical protein	Hypothetical	10500.900	2.338449	0.290544	4.6066	4.09E-06	6.62E-05
C7Y58_08370	FN1069		type I DNA topoisomerase	DNA Replication	13089.212	2.271749	0.294765	4.3144	1.60E-05	2.21E-04
C7Y58_10000	FN1400		serine/threonine protein kinase	Metabolism	775.53071	2.251282	0.239001	5.2354	1.65E-07	3.41E-06
C7Y58_03640	FN1839		glycerol kinase	Metabolism	6925.5807	2.126435	0.281310	4.0042	6.22E-05	7.30E-04
C7Y58_05855	FN0559		phospho-sugar mutase	Metabolism	14664.754	2.107080	0.253552	4.3662	1.26E-05	1.78E-04
C7Y58_01095	FN1718	*secA*	protein translocase subunit SecA	Transport	29760.474	2.053965	0.209438	5.0323	4.85E-07	9.21E-06
C7Y58_06330	FN0656		FMN-binding protein	Metabolism	3729.3801	2.041441	0.225789	4.6124	3.98E-06	6.49E-05
C7Y58_09870	FN1373		Cys-tRNA(Pro) deacylase	Metabolism	499.65365	1.976954	0.242051	4.0361	5.43E-05	6.57E-04
C7Y58_06325	FN0655		FMN-binding protein	Metabolism	14049.512	1.950521	0.233196	4.0760	4.58E-05	5.57E-04
C7Y58_01595	FN1797		spermidine/putrescine ABC transporter substrate-	Transport	457.27596	1.931381	0.213453	4.3633	1.28E-05	1.79E-04
C7Y58_09780	FN1354		ABC transporter permease	Transport	3700.3213	-	0.169144	-	6.79E-06	1.05E-04
C7Y58_02660	FN2041		transcription termination/antitermination factor	Transcription	3328.0460	-	0.216417	-	2.54E-05	3.33E-04
C7Y58_04100	FN0205		sodium:glutamate symporter	Transport	11580.680	-	0.199829	-	2.89E-06	4.78E-05
C7Y58_00855	FN1668		choline-phosphate cytidylyltransferase	Metabolism	255.61356	-	0.238158	-	4.11E-05	5.06E-04
C7Y58_00665	FN1629		50S ribosomal protein L6	Translation	12116.002	-	0.234184	-	3.01E-05	3.89E-04
C7Y58_04110	FN0207		2-hydroxyglutaryl-CoA dehydratase	Metabolism	9939.9768	-	0.198755	-	8.47E-07	1.51E-05
C7Y58_00645	FN1625		50S ribosomal protein L15	Translation	2291.8474	-	0.215298	-	3.12E-06	5.13E-05
C7Y58_04120	FN0209		leucine-rich repeat domain-containing protein	Unknown	9009.7671	-	0.212847	-	2.03E-07	4.13E-06
C7Y58_00650	FN1626		50S ribosomal protein L30	Translation	1702.0115	-	0.224443	-	7.98E-07	1.48E-05
C7Y58_09785	FN1355		DUF2318 domain-containing protein	Hypothetical	1178.8397	-	0.225546	-	4.32E-07	8.36E-06
C7Y58_01080	FN1715		ATP-binding protein	Unknown	434.71559	-	0.235062	-	1.00E-06	1.78E-05
C7Y58_00770	FN1651		ABC transporter permease	Transport	185.72864	-	0.302776	-	8.62E-05	9.78E-04
C7Y58_00110	FN1517		leucine--tRNA ligase	Translation	4650.2381	-	0.185515	-	5.82E-11	2.01E-09
C7Y58_01440	FN1789		MATE family efflux transporter	Transport	1324.7486	-	0.285185	-	1.50E-05	2.08E-04
C7Y58_04115	FN0208		2-hydroxyglutaryl-CoA dehydratase	Metabolism	11040.345	-	0.179500	-	2.75E-12	1.18E-10
C7Y58_05590	FN0505		gamma-glutamyl-gamma-aminobutyrate hydrolase	Metabolism	1289.6457	-	0.266078	-	8.34E-07	1.50E-05
C7Y58_02675	FN2038		50S ribosomal protein L10	Translation	4303.5117	-	0.310248	-	2.00E-05	2.65E-04
C7Y58_00640	FN1624		preprotein translocase subunit SecY	Transport	30722.293	-	0.296543	-	7.26E-06	1.11E-04
C7Y58_07995	FN0995		hypothetical protein	Hypothetical	1366.3039	-	0.325967	-	4.44E-05	5.42E-04
C7Y58_02680	FN2037		50S ribosomal protein L7/L12	Translation	5057.8219	-	0.312576	-	1.78E-06	3.02E-05
C7Y58_00805	FN1658		proline--tRNA ligase	Translation	1626.5764	-	0.295144	-	3.74E-07	7.33E-06
C7Y58_05585	FN0504		APC family permease	Transport	2897.7563	-	0.268636	-	1.15E-08	3.03E-07
C7Y58_00050	FN1504		nickel ABC transporter, nickel/metallophore	Transport	6327.2519	-	0.388502	-	7.02E-05	8.19E-04
C7Y58_09605	FN1319		DNA primase	DNA Replication	723.96609	-	0.358955	-	8.76E-06	1.29E-04
C7Y58_08570	FN1110	*dppD*	ABC transporter ATP-binding protein	Transport	525.83181	-	0.388832	-	8.51E-06	1.27E-04
C7Y58_07455	FN0886		TonB-dependent receptor	Transport	2263.1492	-	0.329058	-	6.27E-08	1.44E-06
C7Y58_02090	FN0028		IS1182 family transposase ISFnu2	DNA Replication	531.68074	-	0.347686	-	1.93E-07	3.97E-06
C7Y58_06710	FN0733		peptidase T	Metabolism	1228.6274	-	0.433912	-	1.68E-05	2.30E-04
C7Y58_01075	FN1714		hypothetical protein	Hypothetical	483.30978	-	0.299625	-	3.82E-10	1.21E-08
C7Y58_08030	FN1002		adenosylmethionine--8-amino-7-oxononanoate	Metabolism	1615.5565	-	0.283506	-	1.61E-11	5.89E-10
C7Y58_04965	FN0384		hypothetical protein	Hypothetical	647.24764	-	0.396513	-	1.04E-06	1.83E-05
C7Y58_04805	FN0351		hypothetical protein	Hypothetical	1165.8196	-	0.487047	-	3.61E-05	4.52E-04
C7Y58_04800	FN0350		magnesium transporter	Transport	902.91126	-	0.427586	-	2.50E-06	4.18E-05
C7Y58_02720	FN2029		acetyltransferase	Metabolism	736.87581	-	0.205656	-	7.38E-23	1.08E-20
C7Y58_00760	FN1649	*oppD*	ABC transporter ATP-binding protein	Transport	68.244983	-	0.455510	-	7.39E-06	1.12E-04
C7Y58_07435	FN0882	*hmuV*	ABC transporter ATP-binding protein	Transport	366.11089	-	0.421383	-	1.11E-06	1.93E-05
C7Y58_05075	FN0406	*alr*	alanine racemase	Metabolism	470.03026	-	0.381416	-	6.01E-08	1.40E-06
C7Y58_01525	FN1807		DUF4198 domain-containing protein	Hypothetical	10775.671	-	0.503564	-	3.68E-05	4.58E-04
C7Y58_08130	FN1022		calcium-translocating P-type ATPase, PMCA-type	Transport	977.04585	-	0.354111	-	2.80E-09	7.87E-08
C7Y58_09665	FN1331	*hemK*	protein-(glutamine-N5) methyltransferase, release	Translation	195.74734	-	0.483204	-	1.24E-05	1.75E-04
C7Y58_01540	FN1810	*mntB*	metal ABC transporter permease	Transport	1875.6186	-	0.404598	-	6.29E-08	1.44E-06
C7Y58_04220	FN0229		hypothetical protein	Hypothetical	696.02545	-	0.494095	-	6.34E-06	9.87E-05
C7Y58_00130	FN1521	*dppB*	ABC transporter permease	Transport	2506.6400	-	0.563967	-	7.27E-05	8.39E-04
C7Y58_02880	FN1996	*yhhQ*	transporter	Transport	515.27421	-	0.548087	-	3.58E-05	4.51E-04
C7Y58_04950	FN0381		DUF2194 domain-containing protein	Hypothetical	553.04093	-	0.419984	-	3.00E-08	7.71E-07
C7Y58_02350	FN2105	*tctA*	tripartite tricarboxylate transporter permease	Transport	6118.9554	-	0.422242	-	3.16E-08	8.02E-07
C7Y58_09670	FN1332	*prfA*	peptide chain release factor 1	Translation	2971.1769	-	0.286995	-	3.43E-16	2.52E-14
C7Y58_06050	FN0599		ISL3 family transposase	DNA Replication	107.08896	-	0.445370	-	1.10E-07	2.39E-06
C7Y58_04960	FN0383	*pelF*	lipopolysaccharide N-acetylglucosaminyltransferase	Ultrastructure	314.99762	-	0.477387	-	4.51E-07	8.66E-06
C7Y58_01545	FN1811	*mntA*	metal ABC transporter ATP-binding protein	Transport	1050.2425	-	0.538564	-	7.65E-06	1.15E-04
C7Y58_00930	FN1683		N-acetyltransferase	Metabolism	4921.0118	-	0.532165	-	4.25E-06	6.82E-05
C7Y58_00875	FN1672		MBOAT family protein	Transport	632.98851	-	0.418416	-	1.51E-09	4.48E-08
C7Y58_09680	FN1334	*amiA*	N-acetylmuramoyl-L-alanine amidase	Virulence	2114.9467	-	0.234070	-	2.41E-27	5.50E-25
C7Y58_08575	FN1111		diguanylate phosphodiesterase	Signaling	1025.2506	-	0.388209	-	3.07E-11	1.09E-09
C7Y58_04430	FN0232		DUF2185 domain-containing protein	Hypothetical	121.28615	-	0.468118	-	2.45E-08	6.36E-07
C7Y58_00925	FN1682		lipopolysaccharide biosynthesis protein	Ultrastructure	2399.9059	-	0.474024	-	3.26E-08	8.16E-07
C7Y58_06715	FN0734	*ygiQ*	YgiQ family radical SAM protein	Metabolism	2266.0661	-	0.434534	-	2.37E-10	7.62E-09
C7Y58_00765	FN1650	*oppC*	ABC transporter permease	Transport	103.66348	-	0.537861	-	1.22E-07	2.61E-06
C7Y58_05720	FN0533		putative sulfate exporter family transporter	Transport	1474.17	-	0.686331	-	1.96E-05	2.62E-04
C7Y58_00870	FN1671		ABC transporter ATP-binding protein	Transport	752.57586	-	0.265810	-	5.82E-30	1.50E-27
C7Y58_00025	FN1499		cell surface protein	Virulence	1276.6040	-	0.561608	-	5.54E-08	1.31E-06
C7Y58_05415	FN0469	*cutC*	copper homeostasis protein CutC	Signaling	512.18548	-	0.570438	-	3.79E-08	9.38E-07
C7Y58_04955	FN0382		hypothetical protein	Hypothetical	88.432663	-	0.602243	-	3.96E-08	9.69E-07
C7Y58_00135	FN1522	*dppC*	ABC transporter permease	Transport	1244.0508	-	0.706437	-	1.99E-06	3.36E-05
C7Y58_05715	FN0532		AbrB family transcriptional regulator / Hypothetical	Signaling	1833.2755	-	0.758954	-	1.59E-06	2.71E-05
C7Y58_04225	FN0231		hypothetical protein	Hypothetical	606.49304	-	0.743096	-	2.68E-07	5.35E-06
C7Y58_00145	FN1524	*dppD*	ABC transporter ATP-binding protein	Transport	1608.7082	-	0.304992	-	2.80E-37	1.92E-34
C7Y58_00140	FN1523		diguanylate phosphodiesterase	Signaling	6355.3101	-	0.327872	-	1.62E-34	6.66E-32
C7Y58_00150	FN1525	*dppF*	ABC transporter ATP-binding protein	Transport	2183.4160	-	0.336899	-	2.15E-35	1.10E-32
C7Y58_03500	FN1867	*kdd*	L-erythro-3,5-diaminohexanoate dehydrogenase	Metabolism	10740.475	-	1.178997	-	3.36E-05	4.26E-04
C7Y58_00040	FN1502	*nikC*	peptide ABC transporter permease	Transport	192.81182	-	0.578915	-	5.45E-18	4.67E-16
C7Y58_00045	FN1503	*nikB*	ABC transporter permease	Transport	228.22742	-	0.620921	-	7.50E-17	5.93E-15
C7Y58_00035	FN1501	*nikD*	ABC transporter ATP-binding protein (nickel transport)	Transport	131.75615	-	0.662143	-	2.18E-15	1.44E-13
C7Y58_03495	FN1868	*kce*	endonuclease MutS2+G347	Metabolism	7603.3652	-	1.280523	-	3.84E-05	4.75E-04
C7Y58_00030	FN1500	*nikE*	ABC transporter ATP-binding protein (nickel transport)	Transport	96.410404	-	0.819258	-	7.35E-11	2.44E-09

Supplemental References:

1. Roach, S.N., Shepherd, F.K., Mickelson, C.K., Fiege, J.K., Thielen, B.K., Pross, L.M., Sanders, A.E., Mitchell, J.S., Robertson, M., Fife, B.T. and Langlois, R.A., 2024. Tropism for ciliated cells is the dominant driver of influenza viral burst size in the human airway. *Proceedings of the National Academy of Sciences*, *121*(31), p.e2320303121.
